# Molecular Design, Synthesis and Trypanocidal Activity of Dipeptidyl Nitriles as Cruzain Inhibitors

**DOI:** 10.1371/journal.pntd.0003916

**Published:** 2015-07-14

**Authors:** Leandro A. A. Avelar, Cristian D. Camilo, Sérgio de Albuquerque, William B. Fernandes, Cristiana Gonçalez, Peter W. Kenny, Andrei Leitão, James H. McKerrow, Carlos A. Montanari, Erika V. Meñaca Orozco, Jean F. R. Ribeiro, Josmar R. Rocha, Fabiana Rosini, Marta E. Saidel

**Affiliations:** 1 Grupo de Química Medicinal do IQSC/USP, Instituto de Química de São Carlos, Universidade de São Paulo, São Carlos, São Paulo, Brazil; 2 Faculdade de Ciências Farmacêuticas de Ribeirão Preto, Universidade de São Paulo, Ribeirão Preto, São Paulo, Brazil; 3 University of California San Diego Skaggs School of Pharmacy and Pharmaceutical Sciences, San Diego, California, United States of America; Northeastern University, UNITED STATES

## Abstract

A series of compounds based on the dipeptidyl nitrile scaffold were synthesized and assayed for their inhibitory activity against the *T*. *cruzi* cysteine protease cruzain. Structure activity relationships (SARs) were established using three, eleven and twelve variations respectively at the P1, P2 and P3 positions. A *K*_i_ value of 16 nM was observed for the most potent of these inhibitors which reflects a degree of non-additivity in the SAR. An X-ray crystal structure was determined for the ligand-protein complex for the structural prototype for the series. Twenty three inhibitors were also evaluated for their anti-trypanosomal effects and an EC_50_ value of 28 μM was observed for the most potent of these. Although there remains scope for further optimization, the knowledge gained from this study is also transferable to the design of cruzain inhibitors based on warheads other than nitrile as well as alternative scaffolds.

## Author Summary

Chagas disease is a parasitic infection with high morbidity and mortality that is endemic in much of Latin America where it remains a serious public health problem. With increased migration, Chagas disease represents an emerging worldwide challenge and there is an urgent, unmet need for safe and effective medication. The available drugs to treat Chagas disease may be effective in the acute phase of the disease, but efficacy in the chronic phase remains controversial. They can cause serious side effects that lead sufferers to abandon treatment. Using a hypothesis-driven approach to molecular design and drawing on cysteine protease cruzain structural information, we have mapped structure-activity relationships for a dipeptidyl nitrile scaffold and demonstrated that compounds are competitive inhibitors, bind reversibly and bear trypanocidal activity. The binding mode revealed by the crystal structure of the protein-ligand complex for one of the inhibitors shows that binding involves the formation of a covalent bond between the catalytic cysteine and the nitrile carbon. As such, we believe that our study represents a valuable step in the search for new drugs for the treatment of a neglected disease that continues to affect the lives of millions of people.

## Introduction

Chagas disease is caused by the protozoan parasite *Trypanosoma cruzi*, which is transmitted by blood-sucking reduviid bugs of the subfamily Triatominae [1–3]. Also known as American trypanosomiasis, Chagas disease remains a serious public health problem in Latin America [4, 5] and its spread from non-endemic countries represents an emerging worldwide challenge[6]. While some acute cases may be treated with benznidazole or nifurtimox, both of which show poor side effect profiles [7, 8], there is currently no effective therapy for chronic cases [9, 10]. This has led to the study of many new macromolecular targets and collaborative efforts worldwide, including initiatives such as Drugs for Neglected Diseases (DNDi)[11]. Detection of the parasite remains challenging in the chronic phase of the disease although highly sensitive in vivo imaging that allows parasite burden to be monitored in real time in murine disease models has been reported recently[12].

**Fig 1:**
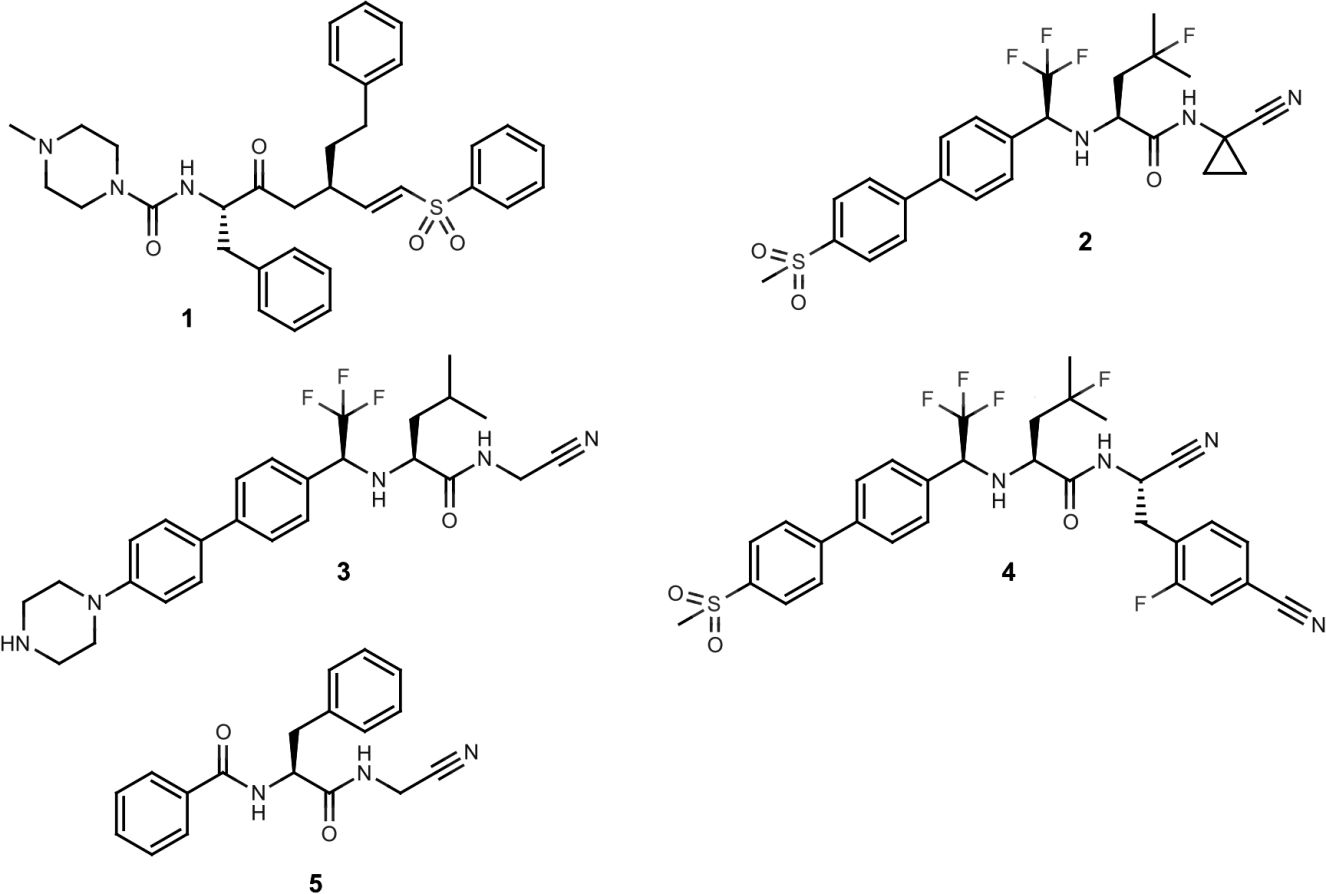
Structures of nitrile-based cysteine protease inhibitors.

The cysteine protease cruzain is considered to be an attractive target for therapeutic intervention in the treatment of Chagas disease [10, 13–17] and the vinyl sulfone K777 (**1**; Fig 1) has been developed as an irreversible inhibitor of this enzyme [15]. The human enzymes most closely related to cruzain are the cathepsins, in particular cathepsin L, and these have been targeted (Fig 1) using the nitrile ‘warhead’ to form a covalent bond with the catalytic cysteine in a reversible manner [18–23]. The cathepsin K inhibitor Odanacatib (**2**; Fig 1) [20] has been evaluated extensively as a treatment for osteoporosis [24, 25], demonstrating that the presence of the electrophilic nitrile [26] in a molecular structure is not necessarily incompatible with achieving pharmacokinetic and toxicological profiles that are compatible with dosing in humans. Structural analogs of **2** have been shown [27] to be potent cruzain inhibitors and *in vivo* activity has been reported for **3** and **4** (Fig 1) in a murine model of acute *T*. *cruzi* infection [28]. Nitrile-based cysteine protease inhibitors targeted at rhodesain have also been shown to inhibit cruzain [29] and a library of cathepsin inhibitors has been used as a source of antiparasitic leads [30].

In the molecular design context, formation of a covalent bond between ligand and target can enable relatively flat or even convex regions on the protein surface to be exploited [31]. The molecular surface of cruzain at the catalytic cysteine is saddle-shaped (i.e. concave in one direction and convex in another) and would not be considered ideal for forming non-covalent interactions. Although it is sometimes assumed that covalently-bound inhibitors are necessarily non-specific, it is important to remember that these ligands also form non-covalent interactions with their target proteins that may modulate affinity. A number of marketed drugs form covalent bonds with their targets and in many cases binding appears to be irreversible [31, 32]. Binding that is irreversible is likely to be advantageous when long residence times [33] are required or if the therapeutic effect depends on inhibition of more than a single target. Potential for immunogenicity [34] is frequently a concern for covalently-bound drugs although a compound that binds irreversibly to intact target may still readily dissociate once the protein has been degraded prior to fragment peptides being presented on MHC class II molecules.

The design objectives of this study were to explore and map structure-activity relationships (SARs) for dipeptidyl nitrile inhibitors of cruzain and to evaluate compounds based on this scaffold for their trypanocidal activity. The dipeptidyl nitrile scaffold, as exemplified by **5** (Fig 1), was adopted for this study since it is an established [22] starting point for synthesis of Papain-like cysteine protease inhibitors and the P2 and P3 substituents can be varied easily using readily available synthetic building blocks. This study builds on previous work by the NEQUIMED team [35] and the molecular design in this study may be regarded as being more hypothesis-driven [36, 37] than prediction-driven in that compounds were selected for synthesis on the basis of their potential to provide information because they had been predicted to be active using quantitative models. It is important to stress that medicinal chemistry design is not just about making predictions and the first step in building a predictive model is to assemble relevant and informative data with which to train the model.

## Materials and Methods

### Ethics statement

The Ethics Committee on Animal Experimentation of the Faculty of Pharmacy of Ribeirao Preto–University of Sao Paulo, approved the cytotoxicity assays (approval no. 010263/2014). This Committee adheres to Conselho Nacional de Controle de Experimentação Animal–CONCEA, created by Brazilian Law number 11794 of 8 October 2008. Assays were run according to the guidelines of the Ministry of Science, Technology and Innovation of Brazil. The Biosafety Committee of the Faculty of Pharmacy of Ribeirao Preto–University of Sao Paulo, also approved the use of genetic modified organisms (approval no. 0019–17).

### Synthetic chemistry

^1^H and ^13^C NMR spectra were recorded on HP – 400 and 500 MHz instruments in CDCl_3_ or (CD_3_)_2_SO with TMS as internal standard. High resolution mass spectra (HRMS) were conducted on a QqTOF Bruker Daltonics spectrometer under the conditions of electrospray ionization (ES), using positive ionization. Infrared spectra were obtained from FT-IR Bomen Hartman & Braun mod MB-102. Melting points were determined on a Quimica Micro MQAPF-302 apparatus and are uncorrected. Thin layer chromatography was performed on Fluka Analytical Sigma-Aldrich silica gel matrix, pre-coated plates with fluorescent indicator 254 nm and/or iodine vapors for detection of amines. Flash column chromatography was performed on silica gel (pore size 60 Å, 70−230 mesh) and eluent hexane/ethyl acetate.

Method development for the characterization and separation of compounds was carried out with a HPLC system consisting of a Shimadzu CBM-20A, degasser DGU-20A_5_, LC-20AT pump, a SIL-20A HT autosampler using a 2000 μL sample loop, a CTO-20A column oven, SPD-M20A detector, and a FRC 10A fraction collector. The detector was set at 200–800 nm. The system was controlled and data analyses were performed using the LC solutions software. Two HPLC protocols were used in this study and, in each case, solvents were filtered through a 0.45 μm Merck-Millipore filter before use and degassed in an ultrasonic bath. In Protocol A, chiral analysis and separation was carried out at 35°C (column oven), using a part of analytical and semi-preparative cellulose-2 Phenomenex column (Analytical: 5 μm, 250 mm x 4.6 mm I.D, semi-preparative: 5 μm, 250 mm x 10 mm I.D), by isocratic elution with a flow rate of 0.5 (analytical) and 2.36 mL min^-1^ (semi-preparative). The mobile phase composition was acetonitrile-water (60:40) (v/v). Volumes of 10 μL (analytical) and 1000 μL (semi-preparative) were injected. Quantification was carried out at 200–800 nm and the chromatographic run time was 20 min. In Protocol B, purity analysis and separation were carried out at 35°C (column oven) using an analytical column Luna c8 (10 μm, 150 mm x 4.6 mm I.D), by isocratic elution with a flow rate of 0.5 mL min^-1^. The mobile aqueous phase composition was 50 to 70% of methanol. All solvents were filtered through a 0.45 μm Merck-Millipore filter before use and degassed in an ultrasonic bath. Volumes of 10 μL to 100 μL were injected. Quantification was performed by measuring sample absorbance at 200–800 nm. The chromatographic run time was 20 min.

**Fig 2:**
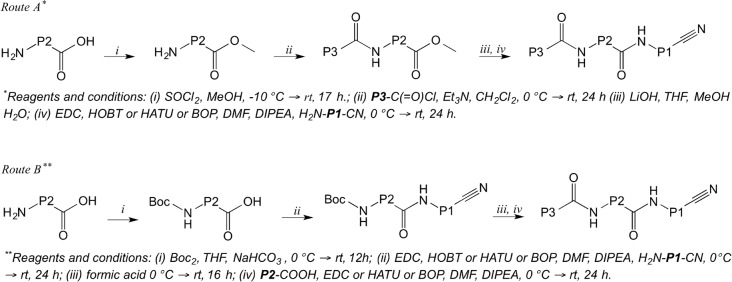
Routes used for the synthesis of the dipeptidyl nitrile cruzain inhibitors.

**Fig 3:**
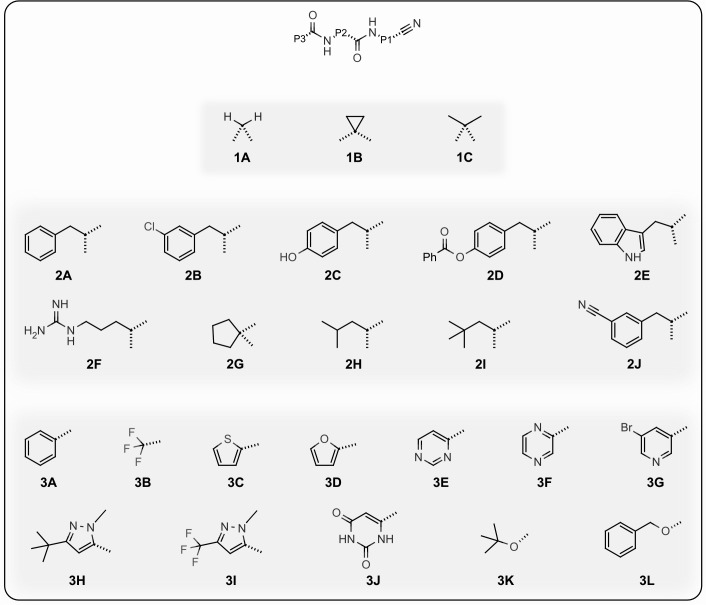
Structural variations at P1, P2 and P3 for dipeptidyl nitrile cruzain inhibitors.

The cruzain inhibitors described in this study were prepared using one of the two routes summarized in Fig 2. Structural variations at the P1, P2 and P3 positions are shown in Fig 3 and the structures of the inhibitors are defined in Table 1.

**Table 1:** Inhibition of cruzain by dipeptidyl nitriles.

Compound^a^	P1 ^b^	P2 ^b^	P3 ^b^	Configuration ^c^	pK_i_ ^d^	Uncertainty ^e^
**5**	1A	2A	3A	S	6.3	0.03
**6**	1A	2A	3A	R	5.2	0.17
**7**	1B	2A	3A	S	6.6	0.03
**8**	1C	2A	3A	S	5.5	0.02
**9**	1A	2B	3A	S	6.6	0.02
**10**	1A	2C	3A	S	6.7	0.02
**11**	1A	2D	3A	S	5.9	0.03
**12**	1A	2E	3A	S	6.4	0.07
**13**	1A	2F	3A	S	3.9	0.02
**14**	1A	2G	3A	N/A ^f^	5.1	0.02
**15**	1A	2A	3B	S	5.4	0.04
**16**	1A	2A	3C	S	6.1	0.02
**17**	1A	2A	3D	S	6.3	0.06
**18**	1A	2A	3E	S	5.9	0.02
**19**	1A	2A	3F	S	5.8	0.04
**20**	1A	2A	3G	S	6.6	0.02
**21**	1A	2A	3H	S	7.2	0.03
**22**	1A	2A	3I	S	6.3	0.05
**23**	1A	2A	3J	S	5.9	0.03
**24**	1A	2A	3L	S	6.3	0.02
**25**	1A	2A	3K	S	5.6	0.03
**26**	1A	2B	3K	S	5.8	0.02
**27**	1A	2H	3K	S	5.9	0.02
**28**	1A	2B	3H	S	7.8	0.03
**29**	1A	2H	3H	S	7.2	0.02
**30**	1B	2H	3H	S	7.6	0.02
**31**	1B	2H	3K	S	6.2	0.02
**32**	1B	2I	3A	S	4.6	0.03
**33**	1B	2A	3I	S	6.4	0.02
**34**	1B	2A	3H	S	7.2	0.03
**35**	1B	2A	3F	S	6.3	0.03
**36**	1B	2J	3A	S	5.4	0.05
**37**	1B	2G	3A	N/A ^f^	4.9	0.02

^a^ Structure number for compound

^b^ See Fig 2

^c^ Configuration of P2 amino acid

^d^ pK_i_ = log_10_(K_i_/M)

^e^ Uncertainty in pK_i_

^f^ Not applicable

Route A (Fig 2) is exemplified by the synthesis of **5**. Compounds **7**, **8**, **9**, **10**, **11**, **12**, **13**, **14**, **20**, **21**, **22**, **28**, **29**, **30**, **32**, **34**, and **37** were prepared in a similar manner.

#### (S)-phenylalanine methyl ester hydrochloride

Thionyl chloride (3.6 equivalents, 181.62 mmol, 12.86 mL) was added dropwise to a solution of (S)-phenylalanine (1 equiv, 10 g, 60.54, mmol) in dry methanol (22 mL) at -10°C and the reaction mixture was allowed to warm to room temperature and then boiled under reflux overnight. The reaction mixture was evaporated under reduced pressure at 40°C, washed with diethyl ether, filtered and dried in a vacuum desiccator to give a white solid (4.68 g, yield 36%) that was used without further purification. M.P. 152–153°C (Lit.155-162°C, (90) NMR ^1^H (400 MHz, DMSO-*d6*) δ 7.35–7.30 (m, 2H), 7.28 (d, *J* = 7.0 Hz, H), 7.23 (d, *J* = 6.7 Hz, 2H), 4.22 (dd, *J* = 7.3, 6.0 Hz, H), 3.64 (s, 3H), 3.20 (dd, *J* = 14.0, 5.8 Hz, 1H), 3.09 (dd, *J* = 14.0, 7.5 Hz, H).

#### (S)-N-benzoyl-phenylalanine methyl ester

Benzoyl chloride (1.1 equiv, 17.85 mmol, 2.07 mL) was added dropwise to a stirred solution of L-phenylalanine methyl ester hydrochloride (1 equiv, 3.5 g, 16.23 mmol) and triethylamine (2.2 equiv, 35.70 mmol, 4.95 mL) in dry CH_2_Cl_2_ (150 mL) at 0°C. The reaction mixture was allowed to warm to room temperature and stirred until starting material was undetectable by TLC. The reaction mixture is washed with 1 M HCl (100 mL), sat. NaHCO_3_ (100 mL) and dried over anhydrous Na_2_SO_4_. The solvent is evaporated under vacuum to afford the crude product as a white solid (3.6 g, yield: 78%), which was purified by column chromatography (silica gel, hexane/ethyl acetate). M.P. 80–81°C (Lit. 81–83°C.). NMR ^1^H (400 MHz, DMSO) δ 3.13 (ddd, *J* = 23.8 13.8 7.7 Hz, 2H), 3.63 (s, 3H), 4.67 (ddd, *J* = 10.0 7.9 5.4 Hz, 1H), 7.18 (t, *J* = 6.6 Hz, 1H), 7.32–7.23 (m, 4H), 7.45 (t, *J* = 7.4 Hz, 2H), 7.45 (t, *J* = 7.4 Hz, 2H), 7.52 (t, *J* = 7.3 Hz, 1H), 7.83–7.74 (m, 2H), 8.83 (d, *J* = 7.8 Hz, 1H).

#### Synthesis of lithium (S)-N-benzoyl-phenylalanine salt

Lithium hydroxide monohydrate (3.3 equiv, 256.5 mg, 10.71 mmol) was added in one portion to ester (1 equiv, 1 g, 3.57 mmol) in THF (10 mL), methanol (5 mL) and water (5 mL) at 20°C. The resulting solution was stirred at 20°C for 4 hours and then concentrated to yield crude lithium salt and dried in a vacuum desiccator for 12 hours. This material was used in the next step without further purification, assuming a quantitative yield. NMR ^1^H (400 MHz, DMSO) δ 3.04 (dd, *J* = 13.3 6.6 Hz, 1H), 3.21 (dd, *J* = 13.3 4.8 Hz, 1H), 4.28 (dd, *J* = 11.7 6.6 Hz, 1H), 7.13–7.06 (m, 1H), 7.20–7.14 (m, 4H), 7.41 (t, *J* = 7.3 Hz, 2H), 7.48 (t, *J* = 7.2 Hz, 1H), 7.71–7.66 (m, 2H), 7.73 (d, *J* = 7.0 Hz, 1H).

#### (2S)-N-(cyanomethyl)-3-phenyl-2-(phenylformamido)propanamide (5)

Aminoacetonitrile hydrochloride (180 mg, 1.17 mmol, 1.6 equiv.) was added to the lithium salt (200 mg, 0.73 mmol, 1 equiv.), BOP (389 mg, 0.88 mmol, 1.2 equiv.) and diisopropylamine (0.5 mL, 2.92 mmol, 4 equiv.) in dimethylformamide (1.88 mL) in a nitrogen atmosphere. The solution was stirred at room temperature for 24 hours. Workup was carried out prior to purification: the mixture was diluted with ethyl acetate and washed with water (10 ml x 10 times), 1 M NaOH (10 mL x 2 times), 1N HCl (10 mL x 1 time), concentrated under reduced pressure, and produced a white solid product (0.1126 g, 50%), which was purified by HPLC protocol A 1: t_R_ = 11.7 min. The product M.P. 179–180°C. ^1^H NMR (400 MHz, DMSO-d6) δ 3.02 (dd, 13.6, 10.8 Hz, 1H), 3.14 (dd, 13.7, 4.3 Hz, 1H), 4.17 (d, 5.6 Hz, 2H), 4.70 (ddd, 10.7, 8.4, 4.4 Hz, 1H), 7.17 (t, 7.3 Hz, 1H), 7.26 (t, 7.5 Hz, 2H), 7.34 (d, 7.3 Hz, 2H), 7.45 (t, 7.4 Hz, 2H), 7.52 (t, 7.3 Hz, 1H), 7.84–7.78 (m, 2H), 8.69 (d, 8.3 Hz, 1H), 8.79 (t, 5.5 Hz, 1H). ^13^C NMR (101 MHz, DMSO-d6) δ 27.62, 37.25, 55.22, 117.32, 127.11, 127.49, 128.75, 128.87, 129.35, 132.30, 133.53, 137.24, 168.21, 172.88. IR (KBr) 3299, 3060, 2236, 1673, 1636, 1532, 1326, 693 cm^-1^. HRMS (ESI (+)) Calcd. for [C_18_H_17_N_3_O_2_] 307.1320, observed: 308.1387 [M+H]. [22]). [α]_D_^24^ = -54.50° ± 1.24; SE = 2.28

Route B (Fig 2) is exemplified by the synthesis of **16**. Compounds **15**, **17**, **18**, **19**, **23**, **26**, **27**, **26**, **33**, **35** and **36** were prepared in an analogous manner.

#### Tert-butyl-N-[(1S)-1-[(cyanomethyl)carbamoyl]-2-phenylethyl]carbamete(Boc-Phe-NCCN (25)

Aminoacetonitrile hydrochloride (3.8 g, 41.5 mmol equiv.) was added to a solution of Boc-Phe-OH (10 g, 37.7 mmol), O-(7-Azabenzotriazol-1-yl)-N,N,N',N'-tetramethyluronium hexafluorophosphate (15.7 g, 41.5 mmol) and N-ethyldiisopropylamine (4 ml, 37.7 mmol) in DMF (15 mL). The resulting solution was stirred at room temperature for 16 hours. The reaction mixture was diluted with ethyl acetate (50 ml) and washed with water (10 x 50 mL), 1M NaOH (2 x 50 mL), 1M HCl (1 x 50 mL) and concentrated. The purification was carried out by HPLC protocol A: t_R_ = 11.6 min giving **25 (**Boc-Phe-NCCN) as a white solid (9.2 g 82%). M.P. 131–133˚C. ^1^H NMR (400 MHz, DMSO-d6) δ 8.44 (t, J = 4.8 Hz, 1H), 7.30–7.15 (m, 5H), 4.18 (dd, J = 13.1, 8.2 Hz, 1H), 4.11 (d, J = 5.6 Hz, 2H), 2.99 (dd, J = 13.8, 5.0 Hz, 1H), 2.80 (dd, J = 13.8, 9.5 Hz, 1H), 1.31 (s, 9H). ^13^C NMR. (101 MHz, CDCl_3_) δ 142.88, 131.04, 127.69, 126.21, 120.81, 107.59, 37.36. [22]). [α]_D_^24^ = +9.57° ± 0.87; SE = 9.18.

#### (2S)-2-amino-N-(cyanomethyl)-3-phenylpropanamide amine

A solution of Boc-Phe-NCCN (5.0 g, 16.5 mmol) in formic acid (5 mL) was stirred for 16 h and then concentrated under vacuum to give COO^.^Phe-NCCN as an oil which was used in the next step without further purification: yield 90% [38].

#### (2S)-N-(cyanomethyl)-3-phenyl-2-(thiophen-2-ylformamido)propanamide (16)

*COO∙Phe-NCCN* (0.220 g 0.82 mmol) was added to a solution of thiophene-2-carboxylic acid (0,100 g 0.75 mmol), O-(7-Azabenzotriazol-1-yl)-N,N,N',N'-tetramethyluronium hexafluorophosphate (0.312 g, 0.82 mmol) and N-ethyldiisopropylamine (0.16 mL, 1.5 mmol) in DMF (5 mL). The resulting solution was stirred at room temperature for 16 hours. The reaction mixture was diluted with ethyl acetate and washed with water (10x), 1M NaOH (2x), 1M HCl (1x) and concentrated. Purified by HPLC protocol A t_R_ = 10.3 min, giving **16** as a white solid (0.160 g 70%). M.P. 162–165 ˚C ^1^H NMR (400 MHz, DMSO-d6) δ 8.81 (t, *J* = 5.6 Hz, 1H), 8.72 (d, *J* = 8.4 Hz, 1H), 7.82 (dd, *J* = 3.8, 1.2 Hz, 1H), 7.71 (dd, *J* = 5.0, 1.1 Hz, 1H), 7.33–7.26 (m, 2H), 7.22 (t, *J* = 7.4 Hz, 2H), 7.18–7.10 (m, 2H), 4.61 (ddd, *J* = 10.6, 8.4, 4.5 Hz, 1H), 4.13 (d, *J* = 5.9 Hz, 2H), 3.09 (dd, *J* = 13.7, 4.5 Hz, 1H), 2.95 (dd, *J* = 13.7, 10.6 Hz, 1H). ^13^C NMR (101 MHz, DMSO-d6) δ 172.01, 161.22, 139.24, 137.99, 133.18, 131.18, 129.15, 128.80, 128.22, 127.95, 126.45, 117.54, 54.63, 36.91, 27.27. HRMS (ESI (+)) Calcd. for [C_16_H_15_N_3_O_2_S] 313.374, Found: 336.0784 [M+Na]. [α]_D_^24^ = -35.81° ± 0.45; SE = 1.27

#### (2S)-N-(1-cyanocyclopropyl)-3-phenyl-2-(phenylformamido)propanamide (7)

Prepared using Route A; yield 60%. Purified by HPLC protocol A: t_R_ = 10.1 min. M.P. 189–190°C. NMR ^1^H (500 MHz, DMSO-*d6*) δ 9.02 (s, 1H), 8.64 (d, *J* = 8.1 Hz, 1H), 7.82 (d, *J* = 7.2 Hz, 2H), 7.52 (t, *J* = 7.3 Hz, 1H), 7.44 (t, *J* = 7.5 Hz, 2H), 7.30 (d, *J* = 7.2 Hz, 2H), 7.25 (t, *J* = 7.5 Hz, 2H), 7.17 (t, *J* = 7.2 Hz, 1H), 4.60 (ddd, *J* = 9.6, 8.2, 5.4 Hz, 1H), 3.03 (ddd, *J* = 23.4, 13.6, 7.6 Hz, 2H), 1.46 (d, *J* = 2.7 Hz, 2H), 1.04 (d, *J* = 9.7 Hz, 2H). NMR ^13^C (126 MHz, DMSO-*d6*) δ 173.18, 166.71, 138.29, 134.26, 131.81, 129.61, 128.61, 128.55, 127.93, 126.80, 121.18, 55.05, 37.39, 20.19, 16.15, 16.13. HRMS (ESI (+)) Calcd. [C_20_H_20_N_3_O_2_] 334.1550, Observed: 334.1550 [M+H]. [α]_D_^24^ = -35.05° ± 0.91; SE = 2.61

#### (2S)-N-(1-cyano-1-methylethyl)-3-phenyl-2-(phenylformamido)propanamide (8)

The general procedure (method A) was followed coupling the corresponding peptidomimetic acid, with the hydrochloric salt of 2-amino-2-methylpropanenitrile yield 40%. Which was purified by HPLC protocol A: t_R_ = 11.3 min. M.P. 114–117°C (Lit. 199–200°C). NMR ^1^H (400 MHz, DMSO-d6) δ 8.66 (s, H), 8.60 (d, *J* = 8.3 Hz, H), 7.86–7.78 (m, 2H), 7.51 (d, *J* = 7.3 Hz, H), 7.45 (t, *J* = 7.4 Hz, 2H), 7.36 (d, *J* = 7.1 Hz, 2H), 7.27 (t, *J* = 7.5 Hz, 2H), 7.18 (t, *J* = 7.3 Hz, H), 4.74 (td, *J* = 8.9, 5.9 Hz, H), 3.04 (dd, *J* = 11.5, 6.9 Hz, H), 1.58 (d, *J* = 15.1 Hz, 6H). NMR ^13^C (126 MHz, DMSO-d6) δ 171.74, 167.34, 137.56, 133.72, 132.14, 129.57, 128.82, 128.58, 127.61, 126.97, 121.89, 54.98, 49.00, 46.07, 37.62, 26.64, 26.35. HRMS (ESI (+)) Calcd. [C_20_H_22_N_3_O_2_] 336,1706. Observed: 336.1689 [M+H].

#### (2S)-3-(3-chlorophenyl)-N-(cyanomethyl)-2-(phenylformamido)propanamide (9)

Yield 17%. Purified by HPLC protocol A: t_R_ = 19.4 min, M.P. 176–178°C. ^1^H NMR (400 MHz, DMSO-d6) δ 3.01 (t, 12.2 Hz, 1H), 3.14 (dd, 13.4, 3.2 Hz, 1H), 4.17 (d, 5.1 Hz, 2H), 4.70 (m, 1H), 7.23 (s, 1H), 7.29 (s, 2H), 7.49–7.39 (m, 3H), 7.51 (d, 7.0 Hz, 1H), 7.80 (d, 7.3 Hz, 2H), 8.72 (d, 8.1 Hz, 1H), 8.81 (s, 1H). ^13^C NMR (101 MHz, DMSO-d6) δ 27.55, 36.70, 49.00, 54.73, 117.24, 126.93, 127.41, 127.99, 128.77, 129.13, 130.35, 132.21, 133.27, 133.47, 139.82, 168.08, 172.44. IR (KBr) 3290, 3062, 2365, 1675, 1637,1535, 1333, 693 cm^-1^. HRMS (ESI (+)) Calcd. for [C_18_H_16_ClN_3_O_2_] 341.0931, found: 342.0974 [M+H].

#### (2S)-N-(cyanomethyl)-3-(4-hydroxyphenyl)-2-(phenylformamido)propanamide (10)

Prepared using route A; yield 48%. Purified by HPLC protocol A: t_R_ = 4.50 min. M.P. 209–210°C. NMR ^1^H (500 MHz, DMSO-*d6*) δ 9.15 (s, 1H), 8.74 (t, *J* = 5.6 Hz, 1H), 8.61 (d, *J* = 8.3 Hz, 1H), 7.81 (d, *J* = 7.1 Hz, 2H), 7.52 (t, *J* = 7.3 Hz, 1H), 7.45 (t, *J* = 7.5 Hz, 2H), 7.10 (d, *J* = 8.4 Hz, 2H), 6.62 (d, *J* = 8.4 Hz, 2H), 4.59 (ddd, *J* = 10.4, 8.3, 4.5 Hz, 1H), 4.15 (d, *J* = 6.3 Hz, 2H), 3.00 (dd, *J* = 13.8, 4.4 Hz, 1H), 2.89 (dd, *J* = 13.7, 10.5 Hz, 1H). NMR ^13^C (126 MHz, DMSO-*d6*) δ 172.20, 166.30, 155.75, 133.87, 131.34, 130.02, 128.16, 128.09, 127.47, 117.53, 114.92, 55.09, 39.52, 36.09, 27.17. HRMS (ESI (+)) Calcd. for [C_18_H_18_N_3_O_3_] 324.1343, observed: 324.1353 [M+H]. [α]_D_^24^ = +38.24° ± 4.11; SE = 10.8

#### 4-[(2S)-2-[(cyanomethyl)carbamoyl]-2-(phenylformamido)ethyl]phenyl benzoate (11)

Prepared using route A; yield 54%. Purified by HPLC protocol B: t_R_ = 7.1 min. M.P. 254–256°C. NMR ^1^H (400 MHz, DMSO-*d6*) δ 8.90 (t, *J* = 5.6 Hz, 1H), 8.77 (d, *J* = 8.4 Hz, 1H), 8.13–8.07 (m, 2H), 7.87–7.82 (m, 2H), 7.73 (t, *J* = 7.4 Hz, 1H), 7.59 (t, *J* = 7.7 Hz, 2H), 7.53 (t, *J* = 7.3 Hz, 1H), 7.49–7.40 (m, 4H), 7.18 (d, *J* = 8.5 Hz, 2H), 4.74 (ddd, *J* = 10.8, 8.4, 4.3 Hz, 1H), 4.18 (d, *J* = 6.9 Hz, 2H), 3.18 (dd, *J* = 13.7, 4.3 Hz, 1H), 3.07 (dd, *J* = 13.6, 10.8 Hz, 1H). HRMS (ESI (+)) Calcd. For [C_25_H_22_N_3_O_4_] 428.1605, observed: 428.1616 [M+H]. [α]_D_^24^ = -71.63° ± 2.99; SE = 4.18

#### (2S)-N-(cyanomethyl)-3-(1H-indol-3-yl)-2-(phenylformamido)propanamide (12)

Prepared using route A; yield 42%. Purified by HPLC protocol A: t_R_ = 12.4 min. M.P. 158–159°C. NMR ^1^H (500 MHz, DMSO-*d6*) δ 10.79 (s, 1H), 8.82 (t, *J* = 5.5 Hz, 1H), 8.60 (d, *J* = 8.0 Hz, 1H), 7.83 (d, *J* = 7.1 Hz, 2H), 7.67 (d, *J* = 7.9 Hz, 1H), 7.51 (t, *J* = 7.4 Hz, 1H), 7.44 (t, *J* = 7.5 Hz, 2H), 7.31 (d, *J* = 8.0 Hz, 1H), 7.20 (d, *J* = 2.2 Hz, 1H), 7.05 (t, *J* = 7.0 Hz, 1H), 6.98 (t, *J* = 7.0 Hz, 1H), 4.73 (s, 1H), 4.17 (dd, *J* = 5.5, 3.3 Hz, 2H), 3.25 (dd, *J* = 14.6, 4.5 Hz, 1H), 3.16 (dd, *J* = 14.6, 10.1 Hz, 1H). NMR ^13^C (126 MHz, DMSO-*d6*) δ 172.46, 166.27, 136.05, 133.83, 131.34, 128.12, 127.48, 127.14, 123.68, 120.90, 118.36, 118.26, 117.59, 111.34, 110.21, 54.03, 39.52, 27.22, 27.17. HRMS (ESI (+)) Calcd. [C_20_H_19_N_4_O_2_] 347.1502, found: 347.1505 [M+H]. [α]_D_^24^ = -27.44° ± 0.19; SE = 0.70

#### (2S)-5-carbamimidamido-N-(cyanomethyl)-2-(phenylformamido)pentanamide (13)

Prepared using route A; yield 18%. Purified by HPLC protocol B: t_R_ = 6.2 min. M.P. 117–118°C. NMR H (400 MHz, DMSO-*d6*) δ 8.96 (t, *J* = 5.5 Hz, H), 8.73 (d, *J* = 7.8 Hz, H), 8.33 (d, *J* = 3.0 Hz, H), 8.10 (dt, *J* = 11.5, 5.7 Hz, 2H), 7.98 (d, *J* = 7.0 Hz, 2H), 7.91–7.85 (m, H), 7.53 (dd, *J* = 14.9, 7.4 Hz, 2H), 7.46 (t, *J* = 7.4 Hz, 3H), 4.45 (ddd, *J* = 9.4, 8.0, 5.3 Hz, 1H), 4.12 (d, *J* = 5.6 Hz, 2H), 3.19–3.08 (m, 2H), 1.89–1.78 (m, 2H), 1.64–1.49 (m, 2H). NMR ^13^C (101 MHz, DMSO- *d6*) δ 172.49, 166.57, 157.18, 133.75, 131.40, 128.26, 128.14, 127.73, 117.58, 53.03, 39.52, 28.24, 27.13, 25.42. HRMS (ESI (+)) Calcd. [C_15_H_21_N_6_O_2_] 317.1720, Present: 317.1726 [M+H]. [α]_D_^24^ = +49.37° ± 2.33; SE = 4.73

#### N-(1-[(cyanomethyl)carbamoyl]cyclopentyl)benzamide (14)

Prepared using route A; yield 72%. Purified by HPLC protocol A: t_R_ = 7.8 min. M.P. 165–166°C. NMR ^1^H (400 MHz, DMSO-*d6*) δ 8.42 (s, 1H), 8.26 (t, *J* = 5.6 Hz, 1H), 7.91 (s, 2H), 7.53 (t, *J* = 7.3 Hz, 1H), 7.46 (t, *J* = 7.3 Hz, 2H), 4.03 (d, *J* = 5.7 Hz, 2H), 2.17–2.07 (m, 2H), 2.06–1.97 (m, 2H), 1.67 (dd, *J* = 11.6, 5.7 Hz, 4H). NMR ^13^C (101 MHz, DMSO-*d6*) δ 174.48, 166.41, 134.35, 131.20, 127.96, 127.79, 117.75, 66.48, 39.52, 36.33, 27.70, 24.26. HRMS (ESI (+)) Calcd. [C_15_H_18_N_3_O_2_] 272.1393, found: 272.1390 [M+H].

#### (2S)-N-(cyanomethyl)-3-phenyl-2-(trifluoroacetamido)propanamide (15)

Prepared using route B; yield 63%. Purified by HPLC protocol A: t_R_ = 6.8 min. ^1^H NMR (400 MHz, DMSO) δ 9.74 (d, *J* = 8.4 Hz, 1H), 8.94 (t, *J* = 5.4 Hz, 1H), 7.24 (d, *J* = 1.5 Hz, 4H), 7.17 (dd, *J* = 9.4, 4.5 Hz, 1H), 4.57–4.48 (m, 1H), 4.16 (d, *J* = 5.5 Hz, 2H), 3.09 (dd, *J* = 13.7, 4.4 Hz, 1H), 2.89 (dd, *J* = 13.7, 10.8 Hz, 1H). ^13^C NMR (100 MHz, DMSO) δ 170.70, 137.50, 129.50, 128.62, 127.03, 119.55, 117.80, 117.25, 114.96, 54.86, 36.80, 28.95, 27.67. HRMS (ESI (+)) Calcd. [C_13_H_12_F_3_N_3_O_2_] 299.2485. Observed: 322.07712 [M+Na].

#### (2S)-N-(cyanomethyl)-2-(furan-2-ylformamido)-3-phenylpropanamide (17)

Prepared using route B; yield 76%. Purified by HPLC protocol A: t_R_ = 10.4 min. M.P. 176–179 ˚C. ^1^H NMR (400 MHz, DMSO-d6) δ 8.82 (t, *J* = 5.5 Hz, 1H), 8.54 (d, *J* = 8.4 Hz, 1H), 7.83 (d, *J* = 0.9 Hz, 1H), 7.32–7.21 (m, 4H), 7.20–7.10 (m, 2H), 6.61 (dd, *J* = 3.5, 1.7 Hz, 1H), 4.65 (td, *J* = 10.3, 4.5 Hz, 1H), 4.16 (d, *J* = 5.7 Hz, 2H), 3.11 (dd, *J* = 13.7, 4.4 Hz, 1H), 3.00 (dd, *J* = 13.7, 10.4 Hz, 1H).^13^C NMR (101 MHz, DMSO-d6) δ 171.77, 157.66, 147.27, 145.19, 137.87, 129.07, 128.14, 126.36, 117.46, 113.90, 111.82, 79.17, 53.88, 36.73, 27.21. HRMS (ESI (+)) Calcd. for [C_16_H_15_N_3_O_3_] 297.3086, Found: 298.1185 [M+H]

#### (2S)-N-(cyanomethyl)-3-phenyl-2-(pyrimidin-4-ylformamido)propanamide (18)

Prepared using route B; yield 56% Purified by HPLC protocol A: t_R_ = 10.4 min. M.P. 171–173˚C. ^1^H NMR (500 MHz, DMSO) δ 9.10 (d, *J* = 1.4 Hz, 1H), 8.85 (dd, *J* = 13.0, 4.2 Hz, 1H), 8.73 (dd, *J* = 2.4, 1.5 Hz, 1H), 7.21 (dd, *J* = 8.8, 5.3 Hz, 2H), 7.18–7.12 (m, 1H), 4.76 (td, *J* = 8.8, 5.0 Hz, 1H), 4.16 (d, *J* = 5.6 Hz, 1H), 3.14 (qd, *J* = 13.8, 7.0 Hz, 1H). ^13^C NMR (126 MHz, DMSO) δ 171.58 (s), 163.12 (s), 148.21 (s), 144.58 (s), 143.94 (s), 143.85 (s), 137.80 (s), 129.58 (s), 128.64 (s), 126.92 (s), 117.84 (s), 54.38 (s), 37.36 (s), 28.96 (s), 27.67 (s). HRMS (ESI (+)) Calcd. [C_16_H_15_N_5_O_2_] 309.1225. Present: 332.1128 [M+Na]. [α]_D_^24^ = -53.28° ± 2.89; SE = 5.42

#### (2S)-N-(cyanomethyl)-3-phenyl-2-(pyrazin-2-ylformamido)propanamide (19)

Prepared using route B; Yield 52% Purified by HPLC protocol A: t_R_ = 8.4 min. M.P. 154–156 ˚C. ^1^H NMR (400 MHz, DMSO) δ 9.09 (d, *J* = 1.5 Hz, 1H), 9.04 (s, 1H), 8.85 (d, *J* = 2.5 Hz, 1H), 8.75–8.70 (m, 2H), 7.23–7.11 (m, 6H), 4.66 (td, *J* = 7.9, 6.2 Hz, 1H), 3.07 (dd, *J* = 7.1, 1.7 Hz, 3H), 1.48–1.40 (m, 2H), 1.09–0.95 (m, 3H). ^13^C NMR (101 MHz, DMSO) δ 172.11 (s), 162.92 (s), 148.21 (s), 144.50 (s), 143.88 (s), 143.84 (s), 137.42 (s), 129.63 (s), 128.59 (s), 126.95 HRMS (ESI (+)) Calcd. [C_16_H_15_N_5_O_2_] 309.1225. Present: 332.1123[M + Na]. [α]_D_^24^ = -13.52° ± 0.55; SE = 4.08

#### (2S)-2-[(5-bromopyridin-3-yl)formamido]-N-(cyanomethyl)-3-phenylpropanamide (20)

Prepared using route A: yield 22%. Purified by HPLC protocol A: t_R_ = 11.3 min. M.P. 179–180°C. ^1^H NMR (400 MHz, DMSO-d6, δ ppm) 2,92 (dd 10,70; 13,75 1H0, 3,14 (dd 4,46; 13,79 1H), 4,16 (dd 1,41; 5,59 2H), 4,68 (m 1H), 7,15–7,31 (m 5H), 8,36 (t 2,01 1H) 8,83–8,86 (m 2H), 8,87–8,88 (d 1,87 1H (100 MHz, DMSO-d6, δ ppm) 27,26 36,87 54,77 117,49 119,95 126,48 128,22 129,09 130,94 137,52 137,82 147,19 152,69 163,60 171,57. IR (KBr) 3282, 3064, 2930, 1680, 1634 cm^-1^. HRMS (ESI (+) Calcd. for [C_17_H_15_BrN_4_O_2_] 386.0378, found: 387.0461 [M+H]. [α]_D_^24^ = +6.84° ± 1.61; SE = 23.51

#### (2S)-2-[(3-tert-butyl-1-methyl-1H-pyrazol-5-yl)formamido]-N-(cyanomethyl)-3-phenylpropanamide (21)

Prepared using route A; yield 43%. Purified by HPLC protocol A: t_R_ = 10.9 min, M.P. 160–162°C. NMR ^1^H (400 MHz, DMSO-d6, δ ppm) 8.77 (t 5.54 1H), 8.57 (d 8.37Hz 1H), 7.15–7.29 (m, 5H), 6.75 (s, 1H), 4.59–4.65 (m, 1H), 4.13 (dd, 1.04; 5.59Hz 2H), 3.85 (s, 3H), 1.23 (s, 9H), 2.91–3.10 (m, 2H). NMR ^13^C (100 MHz, DMSO-d6, δ ppm) 27.24, 30.41, 31.62, 36.74, 38.52, 54.05, 103.81, 117.49, 126.39, 128.16, 129.07, 134.97, 158.69, 159.54, 171.75. HRMS (ESI (+)) Calcd. for [C_20_H_25_N_5_O_2_] 367.2008, found 368.2231[M+H]. [α]_D_^24^ = -27.45° ± 0.73; SE = 2.67

#### (2S)-N-(cyanomethyl)-2-[1-methyl-3-(trifluoromethyl)-1H-pyrazol-5-yl]formamido}-3-phenylpropanamide (22)

Prepared using route X; yield 52% Purified by HPLC protocol A: t_R_ = 10.0 min. M M.P. 204–206°C. ^1^H NMR (400 MHz, DMSO) δ 8.95 (d, *J* = 8.5 Hz, 1H), 8.86 (t, *J* = 5.5 Hz, 1H), 7.29 (ddd, *J* = 20.7, 15.5, 11.1 Hz, 5H), 7.21–7.14 (m, 1H), 4.67 (ddd, *J* = 10.7, 8.5, 4.4 Hz, 1H), 4.24–4.12 (m, 2H), 4.00 (s, 3H), 3.17 (dd, *J* = 13.8, 4.4 Hz, 1H), 2.92 (dd, *J* = 13.7, 10.8 Hz, 1H). ^13^C NMR (101 MHz, DMSO) δ 171.73 (s), 158.55 (s), 139.16 (s), 138.78 (s), 138.41 (s), 138.13 (s), 137.11 (s), 129.49 (s), 128.60 (s), 126.85 (s), 122.86 (s), 120.19 (s), 117.85 (s), 106.47 (s), 54.66 (s), 37.23 (s), 27.65 (s). HRMS (ESI (+)) Calcd. [C_17_H_16_F_3_N_5_O_2_] 379.1256. Present: 402.1163 [M + Na]

#### (2S)-N-(cyanomethyl)-2-[(2,6-dioxo-1,2,3,6-tetrahydropyrimidin-4-yl)formamido]-3-phenyl propanamide (23)

Prepared using route B; yield 56%. Purified by HPLC protocol A: t_R_ = 16.4 min. M.P. 262–265 ˚C ^1^H NMR (400 MHz, DMSO-d6) δ 11.28 (s, 1H), 9.12 (d, *J* = 8.3 Hz, 1H), 8.88 (t, *J* = 5.5 Hz, 1H), 7.31–7.24 (m, 4H), 7.23–7.16 (m, 1H), 6.03 (s, 1H), 4.64–4.55 (m, 1H), 4.17 (dd, *J* = 5.6, 1.3 Hz, 2H), 3.13 (dd, *J* = 13.7, 4.3 Hz, 1H), 2.91 (dd, *J* = 13.7, 10.8 Hz, 1H), 2.08 (s, 1H).^13^C NMR (126 MHz, DMSO-d6) δ 171.46, 164.51, 160.59, 151.10, 144.78, 137.97, 129.46, 128.67, 126.96, 117.82, 100.84, 79.60, 55.21, 37.08, 31.13, 27.63. HRMS (ESI (+)) Calcd. for [C_16_H_15_N_5_O_4_] 341.3214, Found: 342.1196 [M+1]. [α]_D_^24^ = -69.83° ± 2.24; SE = 3.20

#### Benzyl N-[(1S)-1-[(cyanomethyl)carbamoyl]-2-phenylethyl]carbamate (24)

Prepared in an analogous manner to **25**; yield 42%. Purified by HPLC protocol A: t_R_ = 10.6 min ^1^H NMR (500 MHz, DMSO) δ 8.77 t, *J* = 5.5 Hz, 1H), 7.64 (d, *J* = 8.5 Hz, 1H), 7.46–7.10 (m, 10H), 4.95 (q, *J* = 12.7 Hz, 2H), 4.23 (ddd, *J* = 10.4, 8.6, 4.6 Hz, 2), 4.14 (d, *J* = 5.6 Hz, 1H), 2.99 (dd, *J* = 13.7, 4.5 Hz, 1H), 2.77 (dd, *J* = 13.7, 10.4 Hz, 1H).

#### tert-butyl N-[(1S)-1-[(cyanomethyl)carbamoyl]-2-phenylethyl]carbamate (26)

Prepared in analogous manner to **25**; yield 54% Purified by HPLC protocol A: t_R_ = 10.0 min. ^1^H NMR (500 MHz, CDCl_3_) δ 7.27–7.17 (m, 1H), 7.09 (d, *J* = 4.3 Hz, 1H), 6.54 (s, 1H), 5.00 (d, *J* = 7.3 Hz, 1H), 4.61 (dd, *J* = 12.5, 5.9 Hz, 1H), 3.21 (dd, *J* = 13.6, 4.8 Hz, 1H), 3.05 (dd, *J* = 13.7, 6.3 Hz, 1H), 1.44 (s, 9H), 1.31 (s, 1H). [α]_D_^24^ = +18.06° ± 1.05; SE = 5.83

#### tert-butyl N-[(1S)-1-[(cyanomethyl)carbamoyl]-3-methylbutyl]carbamate (27)

Prepared in an analogous manner to **25**; yield 62%. Purified by HPLC protocol A: t_R_ = 7.4 min. NMR ^1^H (400 MHz, CDCl_3_) δ 7.33 (s, 1H), 5.04 (s, 1H), 4.14 (d, *J* = 5.4 Hz, 4H), 1.63 (dd, *J* = 15.1, 9.8 Hz, 3H), 1.51 (d, *J* = 9.0 Hz, 1H), 1.43 (s, 12H), 0.95 (s, 1H), 0.92 (dd, *J* = 9.8, 6.2 Hz, 7H) HRMS (ESI (+)) Calcd. [C_13_H_23_N_3_O_3_] 269.34. Present: 392.1632 [M+Na]. [α]_D_^24^ = +36.16° ± 2.44; SE = 6.76

#### (2S)-2-[(5-tert-butyl-2-methylpyrazol-3-yl)formamido]-3-(4-chlorophenyl)-N-(cyanomethyl) propanamide (28)

Prepared using route A; yield 46%. Purified by HPLC protocol A: t_R_ = 9.5 min. M.P. 175–177°C. ^1^H NMR (500 MHz, DMSO-d6, δ ppm) 1.23 (s 9H) 2.90 (dd 10.93; 13.64 1H), 3.11 (dd 4.31; 13.65 1H), 3.85 (s 3H), 4.11 (dd 3.79; 5.55 2H), 4.59–4.64 (m 1H), 6.75 (s 1H), 7.22–7.29 (m 3H), 8,61 (d 8.59 1H), 8.79 (t 5,58 1H). NMR ^13^C (125 MHz, DMSO-d6, δ ppm) 27.68 30.82 32,04 36,72 38,87 54,17 104,25 117,90 126,83 128,25 129,61 130,42 133,10 135,39 140,95 159,16 159,99 171,88. IR (KBr) 3303 3062 2960 1661 1636 cm^-1^. HRMS (ESI (+)) Calcd. for [C_20_H_24_ClN_5_O_2_] 401.1618, found: 402.1677 [M+H].

#### (2S)-2-[(3-tert-butyl-1-methyl-1H-pyrazol-5-yl)formamido]-N-(cyanomethyl)-4 methylpentanamide (29)

Prepared using route A; yield 68%. Purified by HPLC protocol A: t_R_ = 9.1 min. M.P. 179–180°C. NMR ^1^H (500 MHz, DMSO-*d6*) δ 8.70 (t, *J* = 5.6 Hz, 1H), 8.46 (d, *J* = 8.1 Hz, 1H), 6.88 (s, 1H), 4.43 (ddd, *J* = 10.6, 8.1, 4.6 Hz, 1H), 4.12 (dd, *J* = 5.6, 1.4 Hz, 2H), 3.97 (s, 3H), 1.71–1.59 (m, 2H), 1.57–1.50 (m, 1H), 1.25 (s, 9H), 0.91 (d, *J* = 6.4 Hz, 3H), 0.86 (d, *J* = 6.4 Hz, 3H). NMR ^13^C (126 MHz, DMSO-*d6*) δ 172.68, 159.63, 158.66, 134.91, 117.56, 103.88, 59.74, 50.88, 39.52, 31.62, 30.41, 27.16, 24.30, 23.00, 21.16, 14.08. HRMS (ESI (+)) Calcd. [C_17_H_28_N_5_O_2_] 334.2237. Present: 334.2249 [M+H]. [α]_D_^24^ = -10.90° ± 0.24; SE = 2.31

#### (2S)-2-[(3-tert-butyl-1-methyl-1H-pyrazol-5-yl)formamido]-N-(1-cyanocyclopropyl)-4-methylpentanamide (30)

Prepared using route A: yield 62%. Purified by HPLC protocol A: t_R_ = 8.7 min. M.P. 185–187°C. NMR ^1^H (500 MHz, DMSO-d6) δ 8.96 (s, 1H), 8.41 (d, *J* = 8.0 Hz, 1H), 6.87 (s, 1H), 4.36 (ddd, *J* = 10.4, 8.2, 4.8 Hz, 1H), 3.96 (s, 3H), 1.67–1.56 (m, 2H), 1.47 (dd, *J* = 7.4, 4.7 Hz, 3H), 1.24 (s, 9H), 1.13–1.07 (m, 2H), 0.89 (d, *J* = 6.4 Hz, 3H), 0.85 (d, *J* = 6.4 Hz, 3H). NMR ^13^C (126 MHz, DMSO-*d6*) δ 173.45, 159.60, 158.70, 134.97, 120.79, 103.88, 50.81, 39.52, 38.65, 31.63, 30.42, 24.31, 23.00, 21.25, 19.85, 15.70, 15.62.HRMS (ESI (+)) Calcd. [C_19_H_30_N_5_O_2_] 360.2394, observed: 360.2389 [M+H]. [α]_D_^24^ = -3.75° ± 0.38; SE = 10.3

#### tert-butyl N-[(1S)-1-[(1-cyanocyclopropyl)carbamoyl]-3-methylbutyl]carbamate (31)

Prepared using in an analogous manner to **25**; yield 53% Purified by HPLC protocol A: t_R_ = 8.6 min. NMR ^1^H (400 MHz, DMSO) δ 8.80 (s, 1H), 6.91 (d, *J* = 7.9 Hz, 1H), 3.87 (dd, *J* = 13.6, 8.7 Hz, 1H), 1.60–1.50 (m, 1H), 1.46 (dd, *J* = 7.6, 5.5 Hz, 3H), 1.38 (d, *J* = 10.8 Hz, 9H), 1.07 (dd, *J* = 7.7, 5.4 Hz, 1H), 0.85 (dd, *J* = 8.7, 6.7 Hz, 6H). [α]_D_^24^ = +23.03° ± 4.73; SE = 20.5

#### (2S)-N-(1-cyanocyclopropyl)-4,4-dimethyl-2-(phenylformamido)pentanamide (32)

Prepared using route A; yield 54%. Purified by HPLC protocol A: t_R_ = 7.1 min. M.P. 186–187°C. NMR ^1^H (400 MHz, DMSO-*d6*) δ 8.91 (s, 1H), 8.51 (d, *J* = 8.1 Hz, 1H), 7.90 (d, *J* = 7.0 Hz, 2H), 7.53 (t, *J* = 7.3 Hz, 1H), 7.46 (t, *J* = 7.3 Hz, 2H), 4.49 (td, *J* = 9.2, 3.4 Hz, 1H), 1.76 (dd, *J* = 14.2, 9.3 Hz, 1H), 1.63 (dd, *J* = 14.2, 3.4 Hz, 1H), 1.47 (dd, *J* = 8.0, 5.3 Hz, 2H), 1.11 (dd, *J* = 8.1, 5.4 Hz, 2H), 0.91 (s, 8H). NMR ^13^C (101 MHz, DMSO-*d6*) δ 173.96, 165.89, 133.98, 131.29, 128.14, 127.55, 120.78, 50.69, 44.15, 39.52, 30.29, 29.49, 19.91, 15.75, 15.55. HRMS (ESI (+)) Calcd. [C_18_H_24_N_3_O_2_] 314.1863, observed: 314.1866 [M+H]. [α]_D_^24^ = +17.82° ± 0.88; SE = 4.95

#### (2S)-N-(1-cyanocyclopropyl)-2-{[1-methyl-3-(trifluoromethyl)-1H-pyrazol-5-yl]formamido}-3-phenylpropanamide (33)

Prepared using route B; yield 47% Purified by HPLC protocol A: t_R_ = 9.62 min. M.P. 211–212°C. ^1^H NMR (400 MHz, DMSO) δ 9.04 (s, 1H), 8.91 (d, *J* = 8.2 Hz, 1H), 7.37 (d, *J* = 0.5 Hz, 1H), 7.30–7.22 (m, 4H), 7.22–7.14 (m, 1H), 4.58 (ddd, *J* = 9.9, 8.3, 5.3 Hz, 1H), 4.15 (s, 1H), 4.01 (s, 3H), 3.09 (dd, *J* = 13.6, 5.3 Hz, 1H), 2.91 (dd, *J* = 13.6, 10.0 Hz, 1H), 1.47 (q, *J* = 5.3 Hz, 2H), 1.11–0.98 (m, 2H). ^13^C NMR (101 MHz, DMSO) δ 172.39 (s), 158.47 (s), 139.15 (s), 138.78 (s), 137.86 (s), 137.11 (s), 129.55 (s), 128.58 (s), 126.89 (s), 121.06 (s), 106.51 (s), 54.52 (s), 37.32 (s), 20.17 (s), 16.09 (s). HRMS (ESI (+)) Calcd. [C_19_H_18_F_3_N_5_O_2_] 405.1413. Present: 428.1310 [M +Na]. [α]_D_^24^ = -7.73° ± 0.61; SE = 7.90

#### (2S)-2-[(5-tert-butyl-2-methylpyrazol-3-yl)formamido]-N-(1-cyanocyclopropyl)-3-phenylpropanamide (34)

Prepared using route A; yield 21.6%. Purified by HPLC protocol a: t_R_ = 8.1 min. M.P. 182–183°C. ^1^H NMR (400 MHz, DMSO-d6, δ ppm) 1.05–1.11 (m, 4H), 1.24 (s, 9H), 2.89–3.09 (m, 2H), 3.87 (s, 3H), 4.50–4.56 (s, 1H), 7.21–7.38 (m, 4H), 8.31 (s, 1H), 8.57 (d, 8.59Hz, 1H), 8.57 (d, 8.59Hz, 1H), 9.02 (s, 1H) (100 MHz, DMSO-d6, δ ppm) 15.63 15.68 19.77 30.38 31.60 36.39 36.43 53.64 79.16 103.83 120.60 127.86 129.96 132.64 134.94 140.27 158.69 158.45 172.11. IR (KBr) 3259, 3047, 2960, 1681, 1641 cm^-1^. HRMS (ESI (+) Calcd. for [C_22_H_27_N_5_O_2_] 428.1853, found: 428.1868 [M+H]. [α]_D_^24^ = +73.82° ± 4.48; SE = 6.07

#### (2S)-N-(1-cyanocyclopropyl)-3-phenyl-2-(pyrazin-2-ylformamido)propanamide (35)

Prepared using route B; yield 44% Purified by HPLC protocol A: t_R_ = 9.4 min. M.P. 162–164 ˚C ^1^H NMR (400 MHz, DMSO) δ 9.09 (d, *J* = 1.5 Hz, 1H), 9.04 (s, 1H), 8.85 (d, *J* = 2.5 Hz, 1H), 8.75–8.70 (m, 2H), 7.23–7.11 (m, 6H), 4.66 (td, *J* = 7.9, 6.2 Hz, 1H), 3.07 (dd, *J* = 7.1, 1.7 Hz, 3H), 1.48–1.40 (m, 2H), 1.09–0.95 (m, 3H).^13^C NMR (101 MHz, DMSO) δ 172.11 (s), 162.92 (s), 148.21 (s), 144.50 (s), 143.88 (s), 143.84 (s), 137.42 (s), 129.63 (s), 128.59 (s), 126.95 (s), 120.99 (s), 54.10 (s), 37.59 (s), 20.15 (s), 16.09 (s), 16.04 (s). HRMS (ESI (+)) Calcd. [C_18_H_17_N_5_O_2_] 335.367. Present: 358.1287 [M + Na]. [α]_D_^24^ = -30.09° ± 2.02; SE = 6.71

#### (2S)-N-(1-cyanocyclopropyl)-3-(3-cyanophenyl)-2-(phenylformamido)propanamide (36)

Prepared using route B: yield 39% Purified by HPLC protocol A: t_R_ = 9.7 min. M.P. 191–194 ˚C. ^1^H NMR (400 MHz, DMSO) δ 9.02 (s, 1H), 8.70 (d, *J* = 8.2 Hz, 1H), 7.79 (ddd, *J* = 21.0, 11.0, 1.3 Hz, 3H), 7.68–7.59 (m, 2H), 7.56–7.41 (m, 4H), 4.63 (ddd, *J* = 10.0, 8.2, 5.3 Hz, 1H), 3.10 (ddd, *J* = 23.7, 13.6, 7.7 Hz, 2H), 1.51–1.43 (m, 2H), 1.11–0.99 (m, 2H). ^13^C NMR (101 MHz, DMSO) δ 172.71 (s), 166.77 (s), 140.04 (s), 134.64 (s), 134.10 (s), 133.22 (s), 131.87 (s), 130.67 (s), 129.78 (s), 128.62 (s), 127.84 (s), 121.08 (s), 119.23 (s), 111.46 (s), 54.59 (s), 36.74 (s), 20.18 (s), 16.13 (s), 16.07 (s). HRMS (ESI (+)) Calcd. [C_21_H_18_N_4_O_2_] 358.401. Observed: 381.1343 [M + Na]

#### N-bracket1-[(1-cyanocyclopropyl)carbamoyl]cyclopentyl}benzamide (37)

Yield 57%. Purified by HPLC protocol A: t_R_ = 8.0 min. M.P. 161–162°C. NMR ^1^H (400 MHz, DMSO-*d6*) δ 8.42 (s, 1H), 8.26 (t, *J* = 5.6 Hz, 1H), 7.90 (d, *J* = 7.0 Hz, 2H), 7.53 (t, *J* = 7.3 Hz, 1H), 7.46 (t, *J* = 7.3 Hz, 2H), 4.03 (d, *J* = 5.7 Hz, 2H), 2.16–2.08 (m, 2H), 2.03 (dd, *J* = 11.7, 6.7 Hz, 2H), 1.71–1.63 (m, 4H). NMR ^13^C (101 MHz, DMSO- *d6*) δ 174.48, 166.41, 134.35, 131.20, 127.96, 127.79, 117.75, 66.48, 39.52, 36.33, 27.70, 24.26. HRMS (ESI (+)) Calcd. [C_17_H_20_N_3_O_2_] 298.1550. Present: 298.1540 [M+H].

### Specific rotation ([α])

Specific rotations ([α]^T^ = α/lc, in deg mL g^−1^ dm^−1^, but reported herein in degrees) were observed at the wavelength 589 nm, the D line of a sodium lamp. T was set to be 24°C. Samples were weighed from 0.2 to 7.5 mg using a precision balance (Sartorius, Model CPA26P) and were fully dissolved in methanol (HPLC grade, Panreac) or dimethyl sulfoxide (Sigma Aldrich) for those that were not completely soluble in methanol. The rotations were measured using a Digital Polarimeter (P2000, Jasco): α = observed rotation in degrees; l = cell path length of 1 decimeter; a standard polarimeter tube of 1.0 dm in length; c = concentration in g mL^-1^. Values were calculated using 5 measurements for each compound.

### X-ray crystallography

The recombinant cruzain was expressed and initially purified as recently described by Lee *et al*. [39]. The 0.5 mg mL^-1^ solution of procruzain [in 100 mM sodium acetate buffer (pH 5.2), 300 mM NaCl] obtained after affinity chromatography on HisTrapFF crude nickel columns (GE Healthcare LifeSciences), was activated by incubation with 5 mM DTT at 37°C water bath during 2 hours. Active cruzain was inhibited with molar excess of compound **5** (dissolved in DMSO) to prevent degradation due self-proteolysis. The lack of proteolytic activity was confirmed via fluorometric assay against the substrate Z-Phe-Arg-AMC (Bachem, Km = 1 μM). The protein was dialyzed overnight at 4°C in 50 mM Tris (pH 7.5) 300 mM NaCl buffer and concentrated to 3 mg mL^-1^ for purification using superdex 200 (10/300) size exclusion chromatography column (GE Healthcare LifeSciences). The fractions with pure cruzain inhibited with compound **5** (highest peak at 34 min.) eluted in 50 mM Tris (pH 7.5) 300 mM NaCl buffer, were collected, concentrated to 10 mg mL^-1^, buffer exchanged in 2 mM Bis-Tris pH 5.8, followed by the overnight incubation with 2.5 mM of compound **5** (at least 5 times more inhibitor than protein in molar ratio, and 3.5% of DMSO on final protein solution). Hanging drops encompassing 576 crystallographic conditions [Joint Center for Structural Genomics (JCSG) screens, I to IV, anions suite and cations suite] were configured using Mosquito Nanoliter Dropsetter (TTP Labtech). Each condition was screened in 1:1 and 2:1 ration between protein solution and mother liquor. Crystals of maximum size were obtained after one month from a precipitating agent of 0.1 M HEPES, 1.2 M K/Na tartrate at pH 7.5. Crystals were flash-cooled in liquid nitrogen after soak in 25% ethylene glycol.

Diffraction was measured at beamline 8.3.1 of the Advanced Light Source (ALS, Lawrence Berkeley Lab, CA) under low temperature conditions (100 K), using Elves[40] to determine the data collection strategy. The crystals obtained were fragile with lower diffraction power and very sensitive to radiation damage. 230 frames were collected with 2 seconds of exposition time and 0.4° of oscillation between frames. Reflections were indexed, integrated and scaled using XDS package [41]. The initial phasing model without water, ligand and heteroatoms used for molecular replacement in Phaser [42] was prepared from the model (PDB entry 3KKU). 5 chains by Asymmetric Unit (ASU) related by non-crystallographic symmetry (NCS) were identified in Phaser with top LLG of 5021 and top TFZ of 44. Phenix Refine [43] and Coot [44] were used for all steps of structure refinement and interactive model building. The model was positioned initially by rigid body refinement and subjected to torsional NCS, secondary structure restraints followed by multiple cycles of individual coordinate and B-factors refinement. The inhibitor molecule (compound **5**) was manually placed and fit to electron density of chain A using Coot. Clear and representative density for the entirety inhibitor was observed at better than 1.5 σ above the noise level. Chain B did not have clear electron density for the inhibitor at the region of the covalent attachment with the catalytic Cys25, and to avoid over-interpretation the inhibitor it was not built in this chain. For chains C, D and E that are less exposed to solvent region, weak and no clear electron density was observed for the inhibitor. B-factors were initially refined isotropically and latter subject to TLS. The geometry and the structure were assessed using Molprobity [45]. There was no outlier in the Ramachandran statistics, with 97.3% of all residues on favored regions. The data collection and refinement statistics are provided in Table 2. Structural coordinates and observed structure factor amplitudes were deposited in the Protein Data Bank under accession code 4QH6.

**Table 2:** Data collection and refinement statistics.

Data collection		Refinement	
Wavelength (Å)	1.116	Resolution (Å)	34.73–3.13 (3.24–3.13)
Space Group	P4_3_2_1_2	R_work_ / R_free_ (%)	20.25 / 24.28
No. of molecules in ASU	5	No. atoms	7983
Cell dimensions		Protein	7960
a, b, c (Å)	137.8, 137.8, 166.5	Ligand	23
α, β, γ (°)	90, 90, 90	B-factors (Å^2^)	74.6
*R*_*merge*_ (%)	12.8 (77.2)^a^	Protein	74.5
CC ½	0.998 (0.858)*	Ligand	83.7
Completeness (%)	99.7 (99.6)	RMS deviations	
I / σ(I)	15.9 (2.92)*	Bond lengths (Å)	0.005
Redundancy	8.2 (8.5)*	Bond angle (°)	0.87
No. of reflections (test set)	27306 (1440)*		

^a^ Values in parenthesis represents the highest resolution shells.

### Enzyme inhibition studies

Enzyme kinetic assays were carried out at 37°C in 200 L of a solution containing 100 mM acetate buffer pH 5.5, 300 mM NaCl, 5 mM DTT (dithiothreitol), 5% v/v DMSO (dimethyl sulfoxide), 0.01% v/v Triton X-100 and 0.15 nM cruzain, using Corning® 96-well black flat bottom microplates. The rate of the reaction was monitored using a Biotek Synergy HT plate reader through the fluorescence emission at 460 nm (excitation at 355 nm) due to the hydrolysis of the substrate Z-Phe-Arg-7-amido-4-methylcoumarin (Z-FR-AMC, Sigma-Aldrich). The enzyme stock aliquot was rapidly thawed at 37°C and kept on ice until activation, in which it was incubated for 20 min. in the assay buffer (100 mM acetate pH 5.5 and 5 mM DTT) followed by additional 2 min. incubation with inhibitors before the reaction was started by the addition of the substrate.

Visual inspection and a pre-reading of plate wells were performed to check for possible precipitation and background fluorescence, respectively. Fluorescence emission spectra were also recorded for all inhibitors, using the same excitation wavelength (355 nm) as for the fluorometric assay. None of the compounds displayed a significant fluorescence signal around 460 nm, the emission wavelength used to monitor the reaction kinetic. Thus, potential inner-filter effects did not have to be taken into account in our experiments. The reaction was started by the addition of varying concentrations of the substrate and the wells monitored for a total of 5 minutes of reaction. The initial velocities of the substrate hydrolysis under first-order reaction were calculated by Gen5 Biotek software based on the linear-regression coefficient from the fit to data of Relative Fluorescence Unit (mRFU) as a function of time (min).

Each experiment was performed in duplicates for each compound being tested. A control measurement in absence of inhibitor (*K*_M_) was carried out for each setup plate and the kinetic affinity constants (*K*_i_) were calculated from non-linear fit of Michaelis-Menten curves to the data of initial velocities as a function of eight different concentrations of the substrate Z-FR-AMC from 30.0–0.8 μM. All inhibitors were evaluated in two different concentrations, which were chosen based on a previous screening (percentage of inhibition) at 1.7 μM substrate (~*K*_M_). Additionally, the affinity constant for compound **5** and for the three most potent inhibitors of the series were calculated using three different concentrations. SigmaPlot (v. 10.0) was employed for the non-linear fit and the kinetic parameters determination.

Reversibility of compound **5** was tested by measuring the recovery of enzymatic activity after a rapid and large dilution of the enzyme–inhibitor complex. Compound was incubated at 4.5 μM (10-fold the IC_50_) with 15 nM cruzain (100-fold the concentration required for the activity assay) for 30 min and this mixture is diluted 100-fold into the reaction buffer containing the enzyme substrate to initiate reaction in same conditions as used for *K*_i_ determination with 1.7 μM of Z-FR-AMC. The progress curve for this sample was then measured and compared to that of a similar sample of enzyme incubated and diluted in the absence of inhibitor. The irreversible inhibitor E-64 (CAS 66701-25-5, Sigma Aldrich) was used as reference.

### Trypanocidal activity assays

The assays against amastigote forms were performed in LLCMK2 cells. Previously, the cells were cultivated in 96-wells microplates (10^3^ cells/well). After 2 hours the CL Brener (B5 clone) or Tulahuen trypomastigotes were added in a 1:10 ratio and incubated for another 2 hours. Then, the wells were washed with PBS to remove the extracellular parasites and the tested compounds were added in the final concentrations of 0.031, 0.125, 0.5, 2.0, 8.0, 32.0, 128 and 512 μM. After this, the microplates were incubated at 37°C, in CO_2_ atmosphere, for 5 days. Then, 10 μL of FluoReporter lacZ/Galactosidase Quantitative Kit (Life Technologies) were added and after 30 minutes the fluorometric reaction was read in a microplate reader (Synergy H1, Biotek) at 386 nm excitation and 448 nm emission. For both assays the percentage of parasite lysis was determined from the following formula: %lysis = 100 –{[(X-PC)/(PC-NC)]x100}, where the optical density values of the samples (X), the positive controls (PC) and negative controls (NC) were used. Culture medium was used as positive control (PC) and medium with 0.6% DMSO (v/v) as negative control (NC). All assays were performed in triplicate with two independent experiments.

### Cytotoxicity assay using mouse fibroblast cells

All compounds were subjected to the MTT colorimetric assay using Balb-c 3T3 clone A31 cells (mouse fibroblast cells) acquired from the Rio de Janeiro Cell Bank (BCRJ code 0047). Inhibition values were determined in the cell-based assay as previously described [46]. Briefly, cells were cultured at 37°C in an atmosphere of 5% CO_2_ using DMEM medium (Cultilab, Campinas-SP, Brazil) supplemented with 3.5 g glucose (Sigma-Aldrich), 1% penicillin/streptomycin solution and 10% FBS (Cultilab). Cells were plated at a concentration of 10^5^ cell/mL in 96-well plates and incubated for 24 h. All compounds were freshly diluted from 50 mM DMSO stock solutions to obtain the final concentration of 250 μM and added to each well by replacing the medium. The cell viability was assessed during 24, 48 and 72 h using the MTT (Sigma-Aldrich) reagent, with an incubation time of 3 h. Formazan crystals were dissolved using a solubility reagent composed of DMSO, glacial acetic acid and extran for 1 h. The readout was obtained using Biotek Synergy HT plate reader at 570 nm. Benznidazole was used as control. This assay was done in quatruplicate in two independent experiments. Statistical analyses were made in GraphPad Prism 5. Dunnett’s multiple comparison tests were performed using benznidazole as the standard compound for the ordinary ANOVA analysis using 95% confidence interval.

## Results and Discussion

The cruzain inhibitors described in this study were prepared using two routes as summarized in Fig 2. Some racemization of the P2 amino acid was observed when using route A and resolution of enantiomers by chiral HPLC became necessary. For this reason, the alternative route B was developed and it is particularly suitable for varying the P3 substituent. The structural variations at the P1, P2 and P3 are shown in Fig 3.

Enzyme inhibition results are presented for the inhibitors in Table 1. Protein crystal structures and SAR for cruzain [47, 48] and structurally-related targets such as cathepsin L [18, 49] provide a framework for the design described in this study. Compound **5** (pK_i_ = 6.3) is the structural prototype for this series and has been previously reported to inhibit a number of cysteine proteases [22]. Replacement of the methylene link between amide and nitrile with cyclopropane (**7**; pK_i_ = 6.6) resulted in a two-fold increase in potency and a small but consistent potency difference of 0.3 to 0.4 log units was observed for five pairs of inhibitors in which the P2 substituent was either phenylalanine or leucine. The P1 cyclopropane is common to a number of cysteine protease inhibitors [19, 20] and may confer metabolic stability by virtue of the relatively high carbon-hydrogen bond dissociation energy of cyclopropane [36]. This structural transformation has also been observed to result in a four to forty-fold increase in aqueous solubility for cathepsin K inhibitors based on a cyclohexane dicarboxamide scaffold [19, 50]. In contrast, geminal dimethyl substitution of **5** at P1 resulted in a six-fold decrease in potency (**8**; pK_i_ = 5.5), which may reflect geometric differences between the two linkers.

While the *R*-enantiomer of **5** (**6**; pK_i_ = 5.2) is, unsurprisingly, a less potent inhibitor of the enzyme, introduction of a 3-chloro substituent to phenylalanine is associated with a two-fold increase in potency (**9**; pK_i_ = 6.6). Compound **36** (pK_i_ = 5.5) with a cyano substituent at the meta position of phenylalanine, synthesized in order to probe the backbone amide hydrogen bond donors of Met68 and Asn69, was an order of magnitude less potent than **7** (pK_i_ = 6.6). These molecular recognition elements have exploited in the design of cathepsin inhibitors [49]. Compounds with tyrosine (**10**; pK_i_ = 6.7), tryptophan (**12**; pK_i_ = 6.4) and arginine (**13**; pK_i_ = 3.9) as the P2 substituent were synthesized on the basis of their potential for interaction with Glu208 since analogous interactions have been observed in crystal structures of protein-ligand complexes [47]. Substitution of leucine for phenylalanine at P2 resulted in increases in pK_i_ ranging from 0.3 to 0.4 units although this replacement was not actually made for the structural prototype **5**. In contrast, this substitution has been reported to lead to decreases of 0.3 and 0.7 log units respectively in pK_i_ for **24** and **25** against Bovine cathepsin L [22]. Using 4-methyl-L-leucine as the P2 amino acid led to **32** (pK_i_ = 4.6) and two compounds (**14**; pK_i_ = 5.1) and (**37**; pK_i_ = 4.9) with cycloleucine as the P2 amino acid were 1.2 and 1.7 log units less potent respectively than their phenylalanine analogs **5** and **7**.

A number of structural variations for the P3 substituent (benzoyl in the structural prototype **5**) were explored. The trifluoracetyl derivative **15** (pK_i_ = 5.4) is an order of magnitude less potent than **5** despite the greater hydrogen bond acidity of the P3 amide NH. Replacement of the P3 phenyl ring of **5** with simple heteroaromatic rings typically results in loss of potency although **16** is equipotent with the structural prototype. Even double aza substitutions (**18**; pK_i_ = 5.9; **19**; pK_i_ = 5.8) are relatively well-tolerated and these observations are consistent with contact between hydrophobic protein surface and the face, rather than edge, of P3 aromatic substituent. Amino-protected synthetic intermediates **24** and **25** were, at best, equipotent in comparison with the series prototype **5**. The P3 phenyl of **5** was replaced with 3-bromopyridine to explore the possibility of halogen bond formation [21] with the backbone carbonyl of Thr59, although the potency of **20** (pK_i_ = 6.6) is comparable with that of the reference, which may be interpreted as circumstantial evidence that the halogen bond does not form. Replacing the P3 phenyl of **5** with 1-methyl, 3-t-butylpyrazol-5-yl resulted in a 0.9 unit increase in pIC_50_ (**21**; pK_i_ = 7.2) and this structural change had been observed to potency enhancement of 1.3 log units against cathepsin L which, like cruzain, has a leucine residue in contact with the P3 substituent [18].

**Fig 4:**
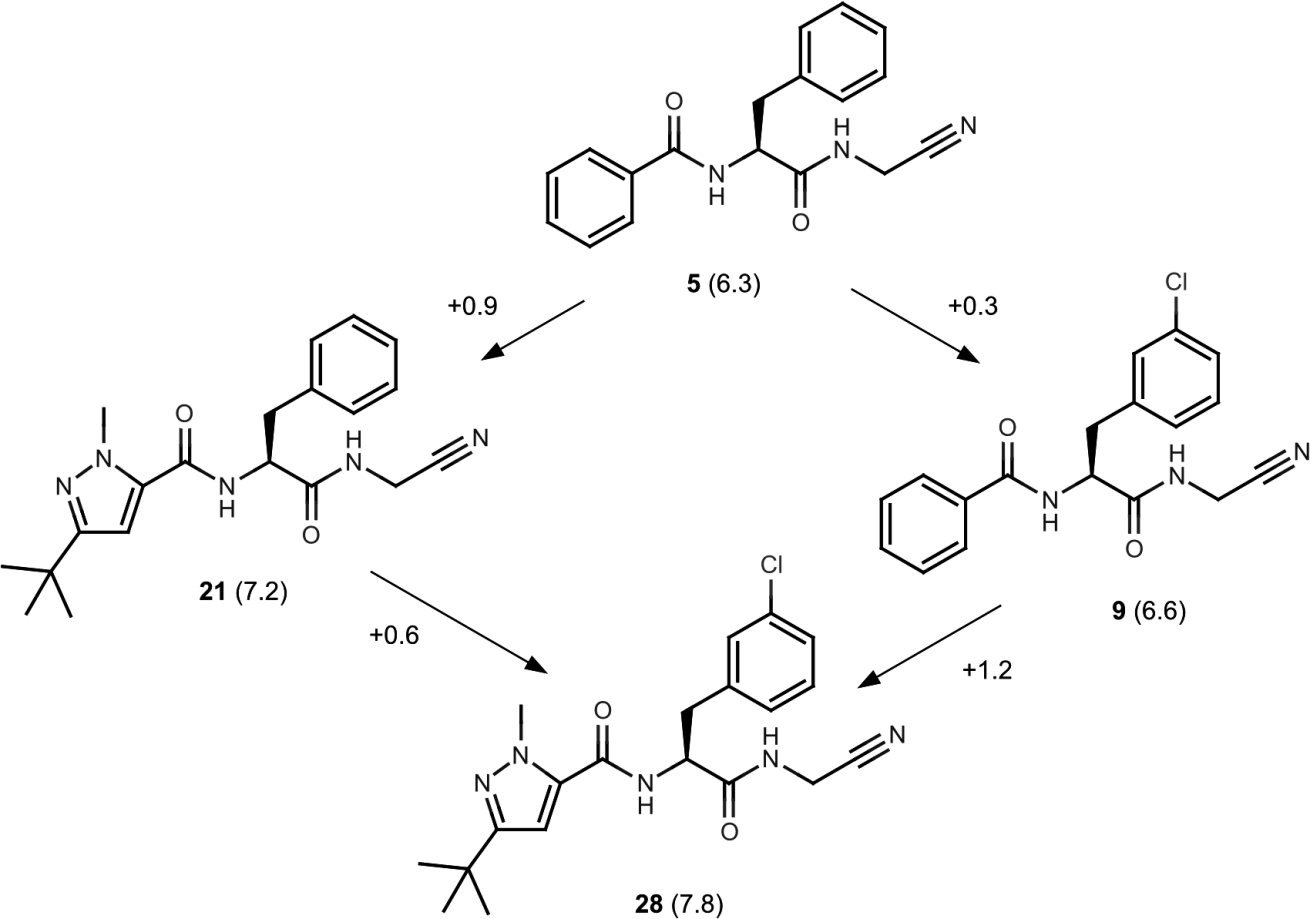
Non-additivity in dipeptidyl nitrile SAR. Values of pK_i_ shown in parentheses and ΔpK_i_ values are associated with the arrows. Differences between ΔpK_i_ values on opposite sides of the ‘square’ reveal mutually reinforcing effects of replacement of the P3 phenyl with 1-methyl, 3-t-butylpyrazol-5-yl and introducing a 3-chloro substituent on the P2 phenyl.

A degree of non-additivity was observed in the SAR and this was quantified using differences (ΔpK_i_) in pK_i_ values. It is important to draw a distinction between a ΔpK_i_ value and the contribution to affinity of an intermolecular contact in a protein-ligand complex which is not, in general, an experimental observable[51]. Non-additive SAR of this nature should be anticipated whenever variable parts of the molecular structure are relatively rigid or are able to influence each other’s orientations with respect to the conserved part of the molecular structure. Fig 4 illustrates how the effects on pK_i_ of replacing the P3 phenyl of **5** with 1-methyl, 3-t-butylpyrazol-5-yl and substituting phenylalanine at C3 with chloro are mutually reinforcing (**28**; pK_i_ = 7.8). Although the effect is small, it is a feature of the SAR that might be exploited, and even enhanced during further optimization of the series.

**Fig 5:**
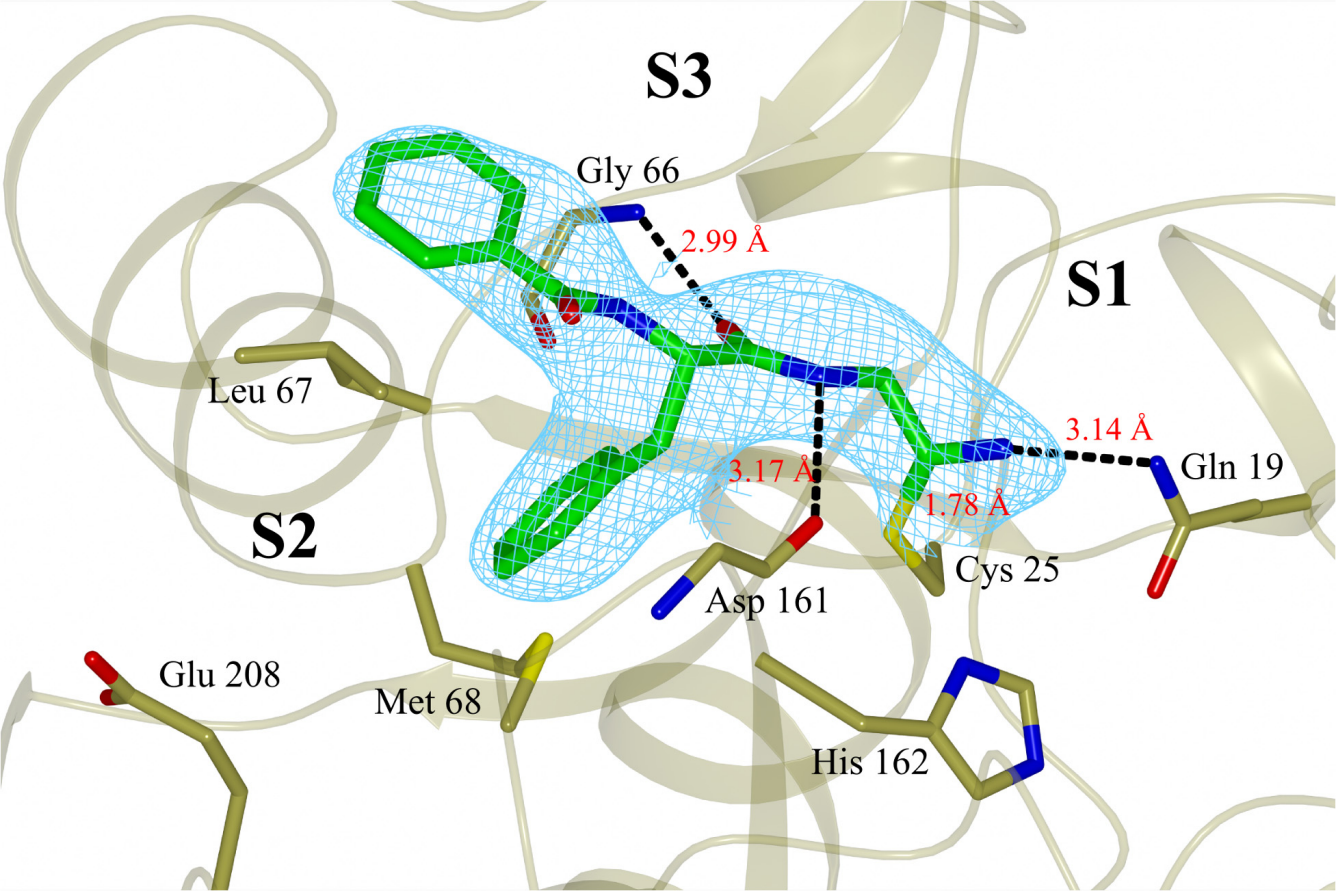
X-ray crystal structure of compound 5 covalently bound to cruzain (chain A), solved to a resolution of 3.13 Å and deposited at PDB with ID code 4QH6. The inhibitor is colored green and the final *2Fo-Fc* electron density map (contoured at 1σ level) is shown in cyan. The catalytic Cys25 and the main residues involved in binding to the inhibitor are also shown. Black dashed lines represent hydrogen bonds. Figure prepared with CCP4mg software [52].

**Fig 6:**
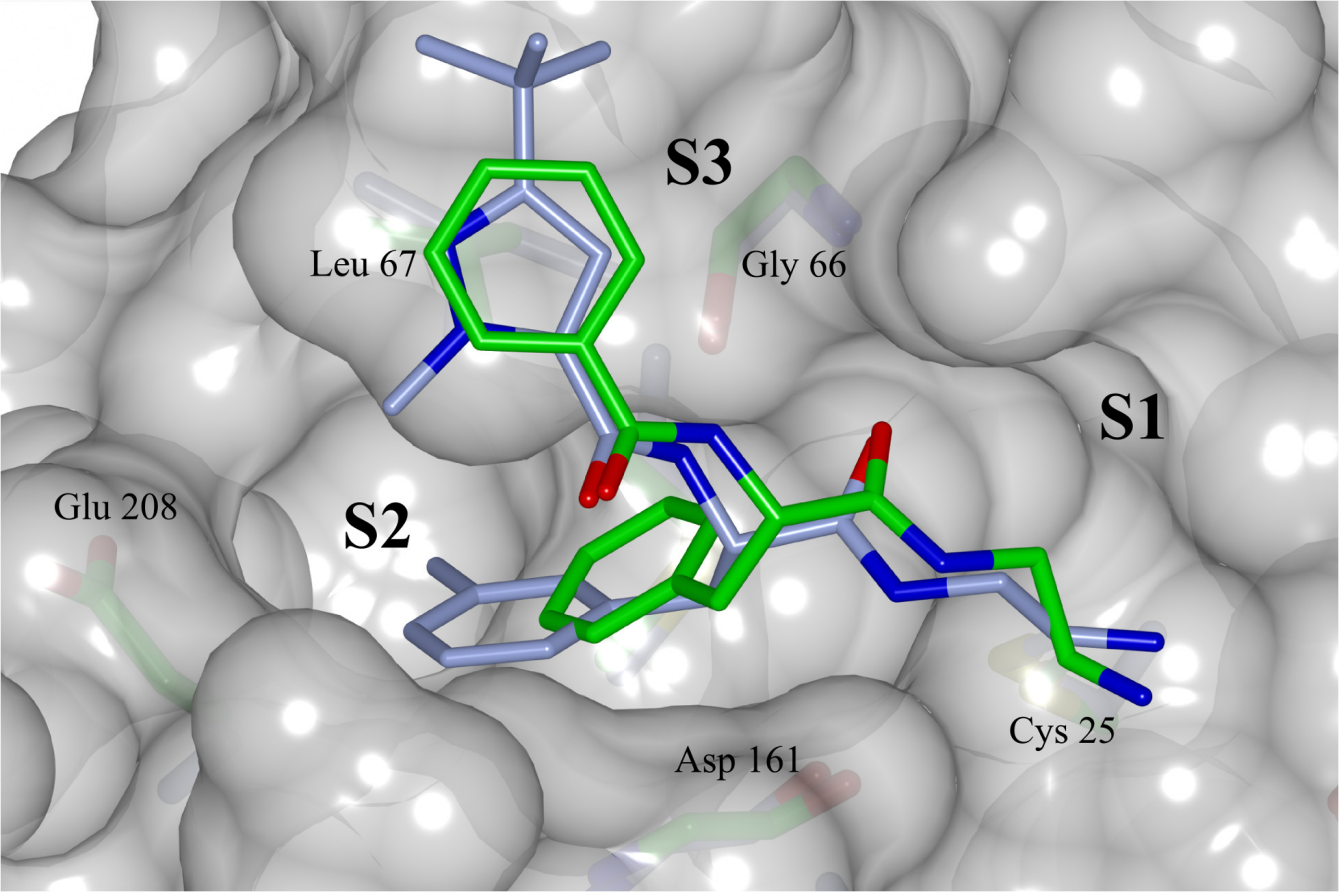
Superposed binding modes of compound 5 and a structural analog bound to cathepsin L (PDB code 3HHA) onto a molecular surface representation of cruzain taken from the crystal structure of 5. Carbon atoms colored green for compound **5** in complex with cruzain and ice blue for cathepsin L analog complex. Oxygen, nitrogen, sulfur colored red, blue and yellow respectively. The binding subsites and the main residues involved at the binding are labeled. Figure prepared with CCP4mg software [52].

The X-ray crystal structure (PDB: 4QH6) determined for the complex of **5** with cruzain reveals a binding mode similar to those observed for structural analogues bound to cathepsin L [18, 49] (Fig 5). The side chain carboxylic acid of Glu208, which corresponds to alanine in cathepsin L, is exposed to solvent. The contact between ligand and Gln19 side chain highlights the need to account for interactions other than the covalent bond when interpreting binding affinity of ligands that bind covalently and reversibly. The ligand phenylalanine group penetrates less deeply into the S2 site than does the corresponding 3-methyphenyl group of an analog bound to cathepsin L (Fig 6). These binding modes hint at a rationale for the non-additivity observed for the effects of the 1-methyl, 3-t-butylpyrazol-5-yl and 3-chlorophenylalanine groups (Fig 4) in that both groups may need to be present for effective penetration of the S2 site.

**Fig 7:**
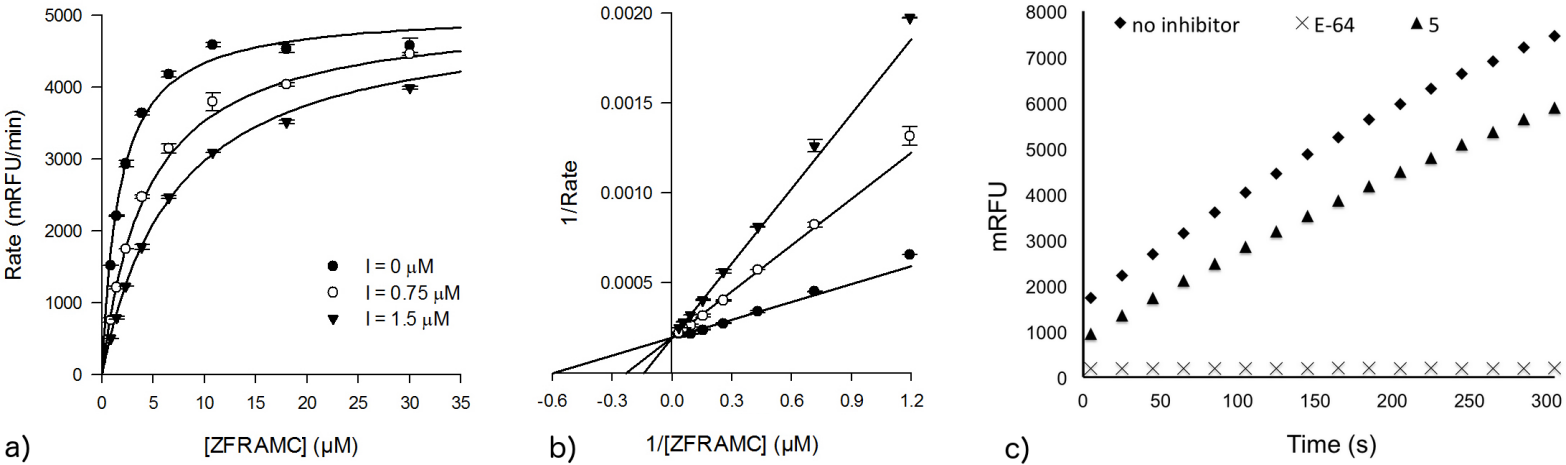
(a) Michaelis-Menten, (b) Lineweaver-Burk and (c) reversibility assay plots compound 5. The effect of increasing concentrations of compound **5** over the rate of reaction shown in (a) and the convergence of double-reciprocal plots over the *y-axis* characterizes compound **5** as a competitive inhibitor relative to the substrate Z-FR-AMC. Additionally, the recovery of enzyme activity after abrupt dilution of a solution containing cruzain and compound **5** after incubation reveals that this compound is acting by a reversible type of inhibition.

The competitive nature and reversibility of inhibition was established for **5** (Fig 7) and a number of the other inhibitors.

**Fig 8:**
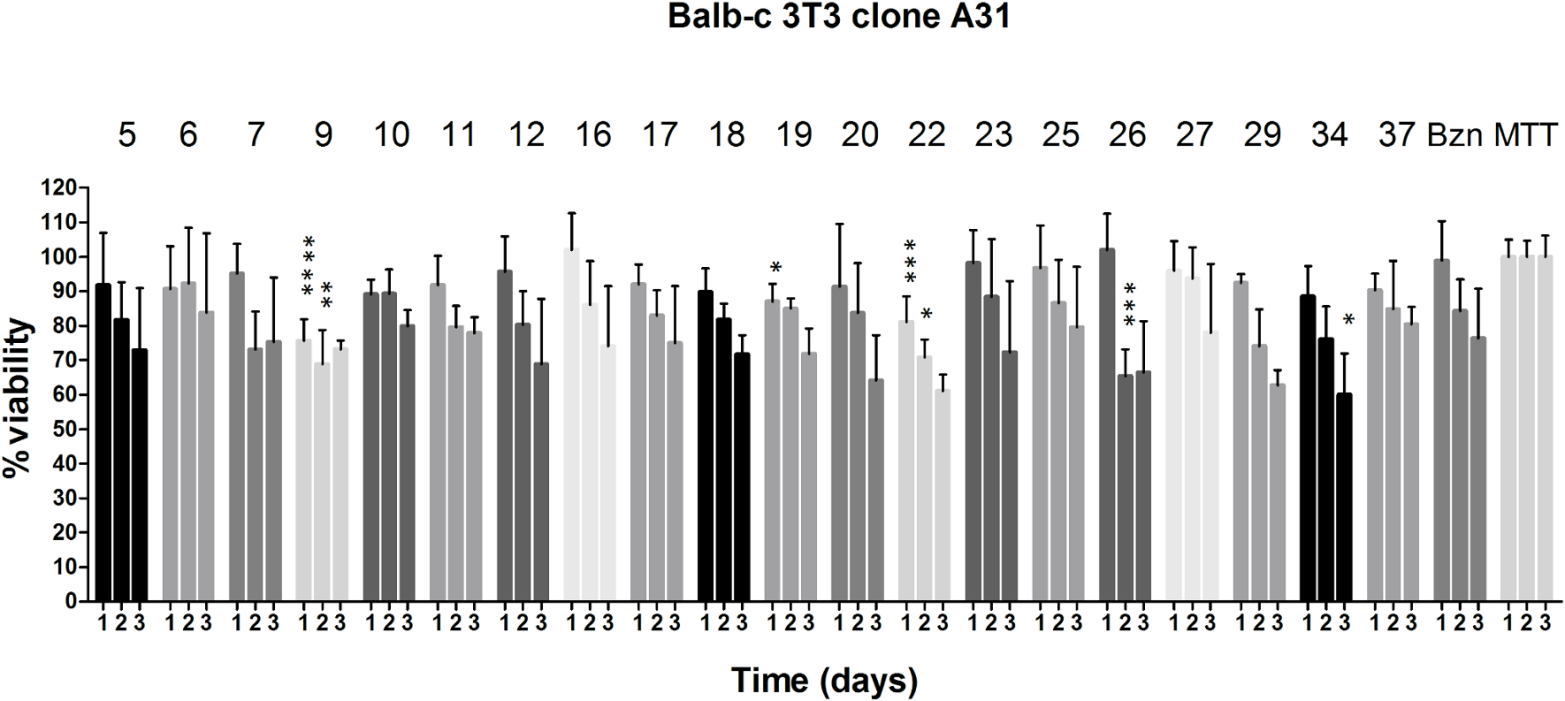
In vitro screening of nitrile derivatives (250 μM) for cytotoxicity using Balb-c 3T3 cell line. One way-ANOVA analysis for each day using benznidazole (Bzn) as control: p < 0.0001 (****), <0.001 (***), < 0.01 (**), < 0.05 (*). MTT is the control for cell growth without the addition of compounds.

A number of compounds were evaluated for their trypanocidal activity against amastigote forms of Tulahuen and CL-Brener *T*. *cruzi* strains and the results are presented in Table 3. No clear relationship emerged between potency of inhibition of the enzyme and trypanocidal activity. A pEC_50_ value of 4.6 was observed for the most potent of these (**12**; pK_i_ = 6.4) indicating that it is an order of magnitude less potent against the Tulahuen strain amastigote than benznidazole (pEC_50_ = 5.8). A pEC_50_ value of 4.3 was observed for **6** which is a relatively weak inhibitor of cruzain (pK_i_ = 5.20) and the possibility must be considered that the weak trypanocidal activity of this compound may be the result of acting on targets other than cruzain. A number of the compounds show % cell lysis at 32 μM comparable to benznidazole against the more resistant CL-Brener strain amastigotes, although this should be seen in the context of the lower activity of benznidazole against this strain. Potential cytotoxicity of inhibitors was assessed with the Balb-c 3T3 cell-based assay and compounds were evaluated over three days using benznidazole as a control. Cytotoxicity at the highest concentration tested that did not lead to precipitation (250 μM) was low for most compounds. The most potent inhibitor of the amastigote *T*. *cruzi* Tulahuen strain (**12**) showed the same range of cytotoxicity when compared to benznidazole (Fig 8).

**Table 3:** Trypanocidal activity of dipeptidyl nitriles against Tulahuen and CL-Brener strains of *T*. *cruzi*.

Compound ID	%cell lysis at 32 μM ^a^ ama Tula	%cell lysis at 32 μM ^b^ ama Brener	pEC_50_ ^a^ama Tula	%cell lysis at 32 μM LLCMK2
**5**	29 ± 1	30 ± 8	3.9	33 ± 2
**6**	41 ± 6	23 ± 3	4.3	46 ± 1
**7**	16 ± 10	12 ± 1	3.9	13 ± 12
**9**	17 ± 2	24 ± 1	4.0	12 ± 1
**10**	32 ± 4	18 ± 1	3.7	18 ± 3
**11**	16 ± 6	17 ± 1	<3.5	8 ± 5
**12**	60 ± 5	17 ± 1	4.6	34 ± 5
**14**	25 ± 4	9 ± 1	3.7	18 ± 1
**16**	30 ± 11	24 ± 2	4.2	19 ± 4
**17**	36 ± 1	21 ± 2	4.0	24 ± 0.2
**18**	17 ± 2	18 ± 1	3.7	20 ± 1
**19**	22 ± 7	21 ± 8	4.1	14 ± 1
**20**	0	25 ± 8	n.d.	17 ± 1
**22**	48 ± 4	20 ± 2	4.0	19 ± 0.4
**23**	0	27 ± 1	n.d.	22 ± 5
**25**	37 ± 1	23 ± 2	4.1	31 ± 2
**26**	26 ± 5	22 ± 1	n.d.	3 ± 2
**27**	0	19 ± 3	n.d.	0
**29**	17 ± 4	10 ± 1	n.d.	27 ± 18
**30**	n.d.	11 ± 6	n.d.	n.d.
**33**	n.d.	22 ± 10	n.d.	n.d.
**34**	0	23 ± 3	n.d.	17 ± 3
**37**	34 ± 5	8 ± 3	4.1	26 ± 3
Benznidazole	78 ± 3	29 ± 2	5.8	31 ± 2

^a^ Amastigote lysis for *T*. *cruzi*—Tulahuen strain.

^b^ Amastigote lysis (%) for *T*. *cruzi*—CL-Brener strain.

*CC_50_ for benznidazole (353±3.70 μM). Not determined (n.d.).

There are a number of reasons that potency in an enzyme kinetic assay may not translate to activity in a cell-based assays. The enzyme inhibitors may be insufficiently permeable or transporter substrates and therefore subject to efflux. Even when an inhibitor accesses the appropriate intracellular compartment, it may still need to compete with high affinity substrates. It is noteworthy that inhibitors with IC_50_ values in the 1–2 nM range (i.e. at least an order of magnitude more potent than the dipeptidyl nitriles described in our study) show activity against an amastigote form of *T*. *cruzi*, that is only in the 5 to 10 micromolar range [28]. Even when the free intracellular concentration of inhibitor is sufficient to engage the proposed therapeutic target, it may still be necessary to perturb other cellular processes in order to achieve the desired phenotypic response. For example, the therapeutic benefits of sorafenib and imatinib appear to derive from the engagement with more than a single target [53]. K777 (**1**) has also shown efficacy in a murine schistosomiasis model [54], demonstrating that it engages targets other than cruzain and the possibility should be considered that its anti-trypanosomal effects may be partly due to inhibition of other cysteine proteases in addition to cruzain.

In conclusion, we have mapped SAR for cruzain inhibition using three, eleven and twelve variations respectively at the P1, P2 and P3 positions of a dipeptidyl nitrile scaffold, determined the binding mode of one of the inhibitors by X-ray crystallography and demonstrated that inhibition is both competitive and reversible. The most potent compound against the parasite was **12**, for which an EC_50_ value of 28 μM was observed and this activity was demonstrated not to be due to general cytotoxicity of the compound. No clear relationship emerged between potency against the enzyme and trypanocidal activity and we have discussed the implications of this for the design of anti-trypanosomal agents. We have shown that the scaffold is of practical value for developing SAR for cruzain inhibitors and there remains scope for further optimization. The knowledge gained from this investigation is also transferable to the design of cruzain inhibitors based on warheads other than nitrile as well as alternative scaffolds.
